# The distribution and evolutionary history of the PRP8 intein

**DOI:** 10.1186/1471-2148-6-42

**Published:** 2006-05-31

**Authors:** Margaret I Butler, Jeremy Gray, Timothy JD Goodwin, Russell TM Poulter

**Affiliations:** 1 Department of Biochemistry, University of Otago, P.O. Box 56, Dunedin, New Zealand

## Abstract

**Background:**

We recently described a mini-intein in the *PRP8 *gene of a strain of the basidiomycete *Cryptococcus neoformans*, an important fungal pathogen of humans. This was the second described intein in the nuclear genome of any eukaryote; the first nuclear encoded intein was found in the *VMA *gene of several saccharomycete yeasts. The evolution of eukaryote inteins is not well understood. In this report we describe additional PRP8 inteins (bringing the total of these to over 20). We compare and contrast the phylogenetic distribution and evolutionary history of the PRP8 intein and the saccharomycete VMA intein, in order to derive a broader understanding of eukaryote intein evolution. It has been suggested that eukaryote inteins undergo horizontal transfer and the present analysis explores this proposal.

**Results:**

In total, 22 PRP8 inteins have been detected in species from three different orders of euascomycetes, including *Aspergillus nidulans *and *Aspergillus fumigatus *(Eurotiales), *Paracoccidiodes brasiliensis*, *Uncinocarpus reesii *and *Histoplasma capsulatum *(Onygales) and *Botrytis cinerea *(Helotiales). These inteins are all at the same site in the PRP8 sequence as the original *Cryptococcus neoformans *intein. Some of the PRP8 inteins contain apparently intact homing endonuclease domains and are thus potentially mobile, while some lack the region corresponding to the homing endonuclease and are thus mini-inteins. In contrast, no mini-inteins have been reported in the VMA gene of yeast. There are several examples of pairs of closely related species where one species carries the PRP8 intein while the intein is absent from the other species. Bio-informatic and phylogenetic analyses suggest that many of the ascomycete PRP8 homing endonucleases are active. This contrasts with the VMA homing endonucleases, most of which are inactive.

**Conclusion:**

PRP8 inteins are widespread in the euascomycetes (Pezizomycota) and apparently their homing endonucleases are active. There is no evidence for horizontal transfer within the euascomycetes. This suggests that the intein is of ancient origin and has been vertically transmitted amongst the euascomycetes. It is possible that horizontal transfer has occurred between the euascomycetes and members of the basidiomycete genus *Cryptococcus*.

## Background

An intein is a specific insertion within a host protein that is excised during protein maturation, that is, post-translationally [1]. This maturation process is termed "protein splicing" and involves precise excision of the internal protein (intein) sequence and ligation of the flanking external protein (extein) sequences to form a peptide bond. The excision of the intein and the subsequent ligation of the flanking host exteins are catalysed by the intein itself [2,3]. The sequence encoding the intein appears as an in-frame insertion within the gene for the host protein. For the sake of simplicity, this intein encoding sequence is also often referred to as an intein.

As well as controlling their own protein splicing, many inteins also include site-specific 'homing' DNA endonucleases. These homing endonucleases belong to several distinct families (for example the LAGLIDADG and His-Cys box groups); the LAGLIDADG type is by far the most common in inteins. Related homing endonucleases are found encoded in Group I self-splicing introns [4,5]. Homing endonucleases can cause a gene conversion event that converts a cell heterozygous for the intein into a homozygote. To initiate this gene conversion, the homing endonuclease, encoded by an intein sequence located at a specific site, recognises a long DNA target sequence corresponding to an unoccupied allelic site. The homing endonuclease generates a double strand chromosomal break within the unoccupied target sequence. This break is repaired by the host DNA repair machinery using as a template the allele containing the intein gene. This gene conversion results in the replication of the intein gene into a specific site in a previously unoccupied allele. The occupied allele is no longer a target for the homing endonuclease because the target site is split by the insertion [6]. Thus, inteins are 'selfish' mobile elements that occupy unique, specific sites in the genome. When they are copied into an empty allele, they are still retained by the donor allele. These characteristics make inteins especially effective tools in phylogenetic analysis of such phenomena as horizontal transfer.

It has been hypothesised, using information derived mainly from studies of the VMA intein in *Saccharomyces*, that at the population level the 'super-Mendelian' replication process caused by the homing endonuclease will increase the frequency of the intein within the gene pool of a sexual species, until eventually the intein may come to fixation [7,8]. The rate of spread through a population will depend upon the host mating system [8]. At fixation there will be no remaining unoccupied alleles. Intra-specific movement of the intein will no longer occur and selection will no longer operate on the homing endonuclease function. In the absence of selection, the homing endonuclease function of the intein will become inactive through random mutation [7]. In agreement with this prediction many yeast VMA inteins have been shown to have non-functional homing endonucleases [9]. It has been suggested that inteins depend on horizontal transmission to species that do not already contain an intein at a specific site to ensure their long-term survival [10,11] and retention of active homing endonuclease. This horizontal transfer between species could be initiated by the encoded homing endonuclease. It has been hypothesised [11] that inteins undergo recurrent cycles of (i) horizontal transmission to new genomes, (ii) fixation in the recipient population by homing, (iii) degeneration of the homing endonuclease gene (HEG) due to the absence of target sequences and (iv) eventual loss by deletion of the whole intein.

About 15% of the known inteins lack an endonuclease domain. These minimal protein splicing elements (mini-inteins) are probably derived from full-length inteins by deletion. Mini-inteins are 130–200 amino acids in length with conserved sequence blocks at each end, while 'full-length' inteins (with a central homing endonuclease domain) are about 360–550 amino acids in length [12]. Both mutagenesis studies [13] and 3-D crystal structures [14] have indicated that the two functions inherent in a mobile intein are largely separate. Thus the endonuclease domain is a discrete region of the intein that has little or no functional or structural overlap with the protein splicing domain [15]. Mini-inteins can be considered as an extreme example of HEG corruption and loss. This loss of homing endonuclease function will prevent interspecific or intraspecific replication by homing, restricting the evolution of mini-inteins and their phylogenetic distribution.

Inteins are found in diverse prokaryotes and a few unicellular eukaryotes. Until recently, the only eukaryote nuclear gene intein described was the VMA intein found in the vacuolar ATPase gene of a number of hemiascomycete yeasts such as *Saccharomyces cerevisiae *[16]. These inteins all appear in exactly the same site (VMA-a) within the host *VMA1 *gene. Such inteins are referred to as 'allelic inteins', even though they are from different species (there is a non-allelic intein in the VMA-b insertion site of the vacuolar ATPase of several Archaea). It was analyses of the yeast VMA inteins, all of which contain a LAGLIDADG HEG, which suggested the possibility of frequent horizontal transfer between species of ascomycete yeasts although the mechanism for such a transfer of nuclear genes is unknown [17,18]. The suggestion was based on the comparison of the intein phylogeny and the host gene (*VMA1*).

Recently we reported the presence and sequences of a second set of allelic nuclear encoded inteins (PRP8 inteins) in a large number of strains of *Cryptococcus neoformans *and in the related species *Cryptococcus gattii *[19,20]. *Cryptococcus neoformans *is a basidiomycete fungus capable of causing serious infections in both immunocompromised and immunocompetent people [21]. *C. neoformans *is divided into two varieties, *C. neoformans *var. *neoformans *and *C. neoformans *var *grubii*. Molecular phylogenetic work indicates that the *grubii *and *neoformans *varieties are separated by ~18.5 million years of evolution, and these varieties diverged from *C. gattii *~37 million years ago [22]. Intein encoding sequences are present in the PRP8 genes of both *C. neoformans *varieties and also in *C. gattii*. These inteins all lack homing endonuclease domains and are thus mini-inteins. The sequence differences of the mini-inteins reflect the relationships among the host species; for example, the variety *neoformans *and variety *grubii *mini-inteins are more similar to each other than either is to the inteins of *C. gattii *[20].

The *Cryptococcus *mini-inteins are encoded within the nuclear gene for PRP8, a highly conserved protein central in the formation of the spliceosome, that coordinates multiple processes in spliceosome activation [23,24]. The protein-splicing component of the PRP8 inteins must remain functional, even after HEG corruption and loss, so that the intein can remove itself from the PRP8 precursor. If the intein is not removed, it is probable that the spliceosome will be non-functional and the fungus will not be able to process introns from messenger RNA. The presence of the mini-intein in both varieties of *C. neoformans *and in *C. gattii *is consistent with their vertical inheritance from their common ancestor but the original source of the intein in *Cryptococcus *remains unclear. The closest relative of *C. neoformans *and *C. gattii*, *Cryptococcus amylolentus*, does not contain a PRP8 intein, nor does a slightly more distant relative, *C. heveanensis *[20]. The only other member of the Tremallales clade known to contain a PRP8 intein is *Cryptococcus laurentii*, a species relatively distantly related to *C. neoformans*. The *C. laurentii *intein is a full-length, HEG-containing, intein [20].

We have investigated the distribution of the PRP8 intein in order to clarify its evolutionary history and, by inference, to better understand the behaviour of inteins in eukaryotes. Previously, PRP8 inteins have been described from four species of *Cryptococcus *[20] and three from filamentous ascomycetes, *Aspergillus nidulans*, *Aspergillus fumigatus *and *Histoplasma capsulatum *[25]. In order to understand intein distribution we have screened the genomes of various fungi for PRP8 inteins. In this work we describe the PRP8 inteins of *Paracoccidioides brasiliensis *and *Uncinocarpus reesii *of the Onygales, *Botrytis cinerea *of the Helotiales and inteins from diverse members of the Sections Fumigati and Clavati of the Eurotiales. Many of these are mini-inteins. This is in contrast to the VMA inteins of yeast, all of which are full-length, although many of their encoded endonucleases are no longer active. In three orders of euascomycete fungi the *PRP8 *genes have been found to encode inteins with homing endonucleases. Within two of these orders we found closely related species that contain mini-inteins. All three orders contain species that do not contain any PRP8 inteins, often species closely related to intein-carrying species. Thus the phylogenetic distribution of these inteins poses some interesting questions. Comparing and contrasting the datasets of the fungal PRP8 and VMA inteins clarifies some of these questions.

## Results

### PRP8 inteins in ascomycete sequence databases

**Figure 1:**
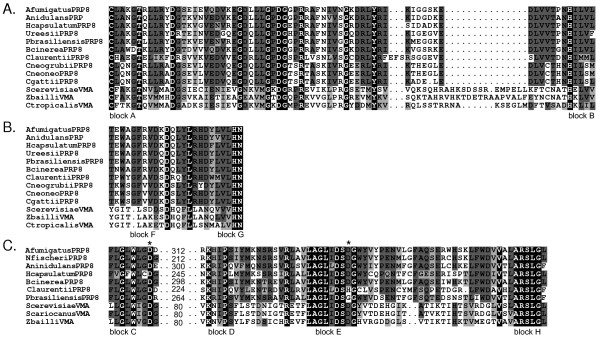
**Insertions in fungal PRP8 genes encode full-length inteins**. The splicing and homing endonuclease domain motifs of ascomycete VMA inteins aligned with PRP8 inteins present in public databases. A. Alignment of the N-terminal splicing domains (blocks A and B; see InBase [62]. B. Alignment of the C-terminal splicing domains (F and G). C. An alignment of the four homing endonuclease domains (blocks C, D, E, H; see InBase) of the full-length PRP8 inteins and the active ascomycete VMA inteins. A region of variable length is indicated between blocks C and D together with the number of residues removed from the alignment. Accession numbers in the NCBI/protein database for the VMA inteins are SceVMA PXBYVA; ScarVMA, CAC86344; ZbaVMA, CAC86348.1; CtrVMA, A46080. Sequences of PRP8 inteins can be found at InBase and/or from accession data in Table 1. The taxonomic relationships of the species are also summarised in Table 1.

To examine the distribution of PRP8 inteins in fungi other than species of *Cryptococcus *we searched the databases of many genome-sequencing projects with the predicted protein sequence of the *C. neoformans *mini-intein CnePRP8. Other searches used as query sequences the PRP8 protein sequences from several fungi. Our searches of the publicly available eukaryote sequence databases revealed the presence of putative full-length PRP8 inteins in the following ascomycete species: *Aspergillus nidulans*, *Aspergillus fumigatus, Paracoccidiodes brasiliensis*, three strains of *Histoplasma capsulatum *and *Botrytis cinerea*. We also detected by this means a mini-intein in *Uncinocarpus reesii*, a close relative of *Coccidioides immitis *and *Coccidioides posadasii*. All of these inteins occur at the same site within the *PRP8 *gene as the inteins in *Cryptococcus*. The insertions all form part of the open reading frame of the 'host' *PRP8 *gene and encode residues at each end that show similarity to the splicing domains of other inteins, especially to the *Cryptococcus *inteins (Figure 1).

The intein encoding sequence in *Paracoccidioides brasiliensis *was represented only by a single, 594bp, EST [GenBank: CN242988]. The first 243bp are predicted to encode the most C-terminal region (81 residues) of an intein similar to the other, complete, ascomycete PRP8 inteins.

*Histoplasma *intein encoding sequences can be found on GenBank accession AAJI01001309, describing data from a NAmI strain, WU24 (from base pair 3014–4618) and on contigs describing two strains which are available at the *Histoplasma capsulatum *genome project website [26]. The sequence encoding HcaPRP8-186AR is found on contig0.41 from position 26003–27604; HcaPRP8-217B is encoded on Histo_FE.contig19 from position 885924–887525. There are 52 variable positions and a single codon indel among the three *Histoplasma *inteins. The *Aspergillus nidulans *intein (AniPRP8) is described in a third party annotation [GenBank: BK001316] referring to the primary data in another accession [GenBank: AACD0100078]. The PRP8 intein of *A. fumigatus *Af293 is encoded by the complementary strand of GenBank: AAHF01000008 (position 782611 to 780155). The *B. cinerea *intein (BciPRP8) is encoded on the complementary strand of supercontig_1.1533, base pairs 22933-20420 [GenBank: AAID01001533]. The mini-intein in *Uncinocarpus reesii *is encoded between positions 33382 to 33921 of GenBank: AAIW01000130; see also Table 1.

**Table 1:** Data describing PRP8 inteins from ascomycetes and basidiomycetes

Intein	Species	Strain	Intein size (aa)	C-termin. Intron	GenBank accession
	Order: Eurotiales				

Ani_PRP8	*Aspergillus nidulans*	FGSC_A4	605	177bp	BK001316
Ani_PRP8	*Aspergillus nidulans*	R20	605	177bp	AY946006
Agi_PRP8	*Aspergillus giganteus*	NRRL6136	167	53bp	DQ285418

	Section Fumigati				

Nfi_PRP8	*Neosartorya fischeri*	FRR0181	517	55bp	AY832922
Afu_PRP8	*Aspergillus fumigatus*	FRR0163	819	51bp	AY832923
Afu_PRP8	*Aspergillus fumigatus*	Af293	819	51bp	XM_744345
Afu_PRP8	*A. fumigatus *var *ellipticus*	NRRL5109	819	51bp	DQ285414
Abr_PRP8	*Aspergillus brevipes*	FRR2439	165	no	AY832924
Avi_PRP8	*Aspergillus viridinutans*	FRR0576	169	no	AY832925
Nsp_PRP8	*Neosartorya spinosa*	FRR4595	169	no	AY832918
Ngl_PRP8	*Neosartorya glabra*	FRR2163	153	no	AY832919
Nfi/Nps_PRP8	*N. pseudofischeri*	FRR0186	169	no	AY832921
Nau_PRP8	*Neosartorya aurata*	NRRL4378	164	no	DQ285415
Nqu_PRP8	*Neosartorya quadricincta*	NRRL4175	169	no	DQ285416
Nfe_PRP8	*Neosartorya fenelliae*	NRRL5534	155	no	DQ285417
					

	Order: Onygales				

Hca_PRP8	*Histoplasma capsulatum*	G217_B	534	no	see text
Hca_PRP8	*Histoplasma capsulatum*	G186_AR	534	no	see text
Hca_PRP8	*Histoplasma capsulatum*	WU24	535	no	AAJI01001309
Ure_PRP8	*Uncinocarpus reesei*	1704	180	no	AAIW01000130
Pbr_PRP8	*Paracoccidiodes brasiliensis*	Pb18	573	no	DQ285419
					

	Order: Helotiales				

Bci_PRP8	*Botrytis cinerea*	B05.10	838	62bp	AAID01001533
					

	Phylum: Basidiomycota				

Cne_PRP8	*Cryptococcus neoformans*	IUM93-3231	172	52bp	AY422973
Cne_PRP8	*Cryptococcus neo. grubii*	PHLS8104	171	53bp	AY422974
Cba_PRP8	*Cryptococcus gattii*	WM02.98	170	65bp	AY422975
Cla_PRP8	*Cryptococcus laurentii*	CBS139	522	no	AY836254

### Detection and sequencing of further PRP8 inteins in ascomycetes

#### Aspergillus/Neosartorya

##### Full-length inteins

We used data from the *Aspergillus *sequence databases to design primers to amplify and sequence PRP8 inteins from other strains of *A. fumigatus *(including FRR0163) and from *A. fumigatus *var.*ellipticus *NRRL5109 [GenBank: DQ285414]. *A. fumigatus *var.*ellipticus *NRRL5109 is a member of a small group of strains that form a cryptic species clade (*A. fumigatus "occultens*" [27]). In addition, we amplified and sequenced a full-length intein from *Neosartorya fischeri *(FRR0181), a species closely related to *A. fumigatus*. We also obtained the intein sequence of *A. nidulans *R20 [GenBank: AY946006]. It is identical to that of the whole genome sequence strain, FGSC_A4; both strains originate from Glasgow University. It should be noted that the genus *Aspergillus *is large and diverse, for example *A. fumigatus *and *N. fischeri *are much more closely related to each other than either is to *Aspergillus *(*Emericella*)*nidulans *[28,29]. *N. fischeri *(FRR0181) is also known as NRRL181 and is now the subject of a genome sequencing project at The Institute for Genomic Research (TIGR). The intein encoding sequence from *A. fumigatus *FRR0163 [GenBank: AY832923] is identical to that of the fully sequenced strain of *A. fumigatus *(Af293), except for one silent third-codon position change in 2457bp (not shown). The intein in *A. fumigatus *var.*ellipticus *NRRL5109 varies from that in *A. fumigatus *(FRR0163) at 12 positions, resulting in four amino acid substitutions in 819 residues. The PRP8 intein of *N. fischeri *[GenBank: AY832922] is 94% identical at the DNA level to the *A. fumigatus *intein. There are three indels of various sizes (609bp, 42bp and 285bp in AfuPRP8) that do not include any of the conserved sequence motifs of the splicing or homing endonuclease domains.

##### Mini-inteins

**Figure 2:**
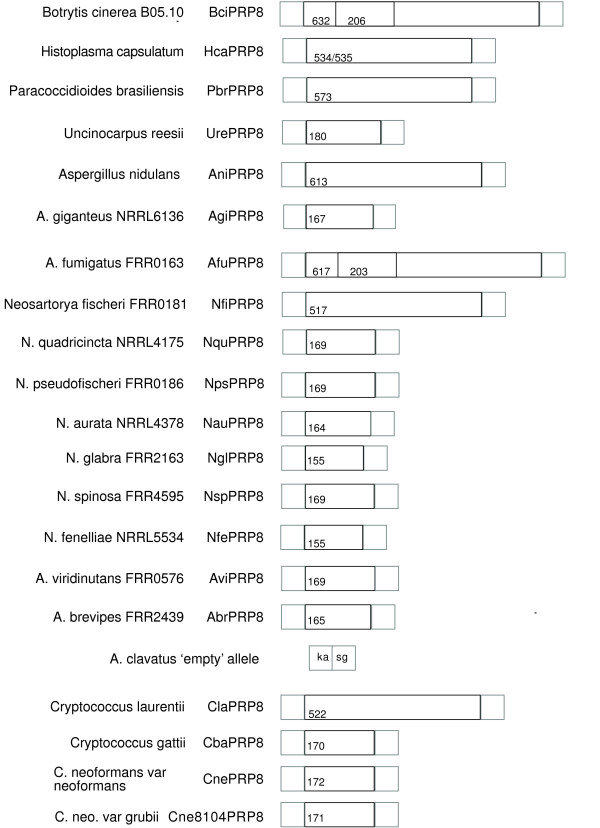
**Full-length and mini-inteins encoded within fungal PRP8 genes, drawn to scale**. Numbers within the boxes represent intein length (amino acid residues); boxes within BciPRP8 and AfuPRP8 contain the extra insertion (206 and 203 residues, respectively) near the end of the N-terminal splicing domain. The taxonomic relationships of the species are summarised in Table 1.

We attempted to amplify PRP8 intein sequences from other species of *Aspergillus *and *Neosartorya *from the section Fumigati (that is, species closely related to *A. fumigatus*) and from more distantly related species using primers complementary to internal intein sequences. These attempts were unsuccessful. In contrast, when we used primers complementary to the PRP8 regions flanking the intein insertion site we detected PCR products, but these were much shorter than would be expected if a full intein were present. These PCR products were sequenced directly, yielding sequences representing mini-inteins in six species of *Neosartorya *(*N. spinosa*, *N. glabra, N. fenelliae, N. quadricincta, N. aurata, N pseudofischeri *FRR0186) and in three species of *Aspergillus *(*A. brevipes, A. giganteus *NRRL 6136 and *A. viridinutans*) (Figure 2) (Table 1). Both *N. spinosa *and *N. glabra *are sometimes described as varieties of *N. fischeri*, but are more distinct from *A. fumigatus *than is *N. fischeri *(Girardin, Monod and Latge 1995). The taxonomy of both *N. glabra *and *N. spinosa *isolates is undergoing revision [30]. Of these nine species with PRP8 mini-inteins, all except *A. giganteus *NRRL 6136 are members of the section Fumigati. *A. giganteus *is a member of the section Clavati [31].*N. pseudofischeri *FRR0186 is the designation we have given to a strain that was originally described as *N. fischeri*. Our analysis of other sequences derived from this strain (ITS and cytB) suggests that it is a member of the *N. pseudofischeri *species. The ITS sequence of FRR0186 is very closely similar (99%) to that of *N. pseudofischeri *[GenBank: AF459729]; the next best match (97% identity) is to *N. fischeri *[GenBank: AF176661]. *N. pseudofischeri *has occasionally been isolated as an opportunistic infection [32]; some isolates have been initially identified as *A. fumigatus *[33].

##### Empty alleles

When using primers complementary to the PRP8 regions flanking the intein insertion site we detected short PCR products from *Aspergillus unilateralis *(FRR0577), *A. clavatus *(NRRL5811) and four strains of *A. lentulus*. These PCR products yielded sequence showing 'empty' alleles; that is, there were no PRP8 inteins present in these strains. *A. lentulus *is a newly recognised species, originally isolated from patients at the Fred Hutchinson Cancer Research Center [34].

#### Paracoccidioides

Using DNA from strain Pb18 of *Paracoccidioides brasiliensis *(a kind gift from Professor Gusatvo Goldman) and a combination of primers, some designed to complement regions of the EST [GenBank: CN242988] that carries a part of a PRP8 intein and other less specific primers designed to complement regions of the *PRP8 *gene, we amplified the whole of the intein-containing PRP8 region from *P. brasiliensis*. The predicted protein sequence reveals an intein containing a homing endonuclease domain; the intein sequence and flanking regions are described in GenBank accession DQ285419. The protein sequence of PbrPRP8 from strain Pb18 has 53% identity with that of the *Histoplasma capsulatum *intein HcaPRP8_217B and 45% identity to the *Aspergillus nidulans *intein AniPRP8. In the 72-residue PRP8 intein region shared by strains Pb18 and Pb01 (GenBank: CN242988), 65 (90%) are identical.

#### Botrytis

We attempted to amplify a PRP8 intein encoding sequence from a strain of *Botrytis cinerea *isolated in New Zealand. The strain was confirmed as belonging to *B. cinerea *by amplifying and sequencing the ITS1 and ITS2 regions of the ribosomal gene array (data not shown). Amplification of the region surrounding the PRP8 intein insertion site, however, showed that this strain does not contain an intein, in contrast to strain B05.10 from the sequence project.

### Protein domains encoded by PRP8 intein sequences

**Figure 3:**
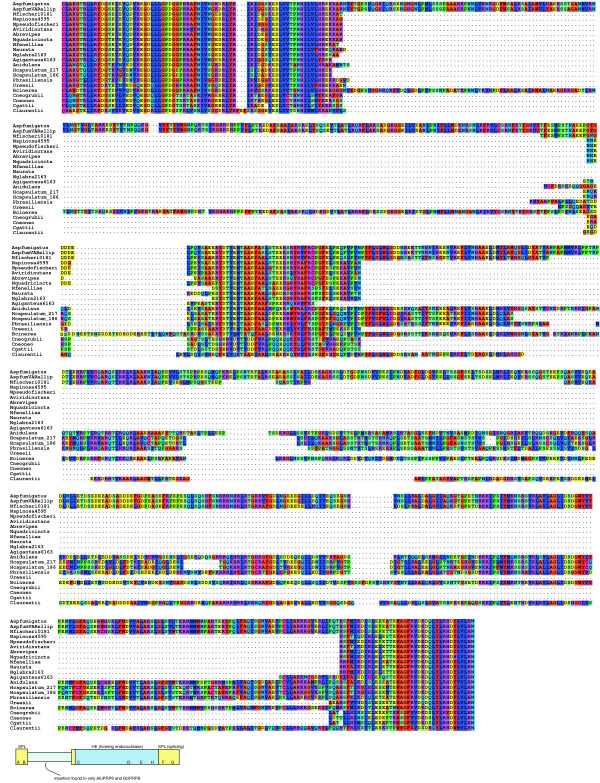
**An alignment of ascomycete and basidiomycete PRP8 inteins**. Similar residue types have the same colour background. Accession numbers for the PRP8 intein sequences are in Table 1. The box diagram at the base of the alignment indicates the general shape of the largest inteins in the alignment. The arrangement of the splicing domains (blocks A, B, F and G) and endonuclease domains (blocks C, D, E and H) are indicated.

The splicing domains and endonuclease domain of PRP8 inteins were predicted through comparison with those of the *Saccharomyces cerevisiae *VMA intein (SceVMA). We identified the conserved sequence blocks using sequences of known inteins held at InBase [12]. There are homing endonuclease domains in the PRP8 inteins of *A. fumigatus*, *A. nidulans*, *N. fischeri*, *H. capsulatum*, *P. brasiliensis *and the genome sequence strain of *B. cinerea*, but none in PRP8 inteins of other *Aspergillus *or *Neosartorya *species (including *N. pseudofischeri *FRR0186). The AfuPRP8 inteins (from FRR0163, from the genome sequencing strain, Af293, as well as from *A. fumigatus *var. *ellipticus*, NRRL5109) include a large insertion of 203 amino acid residues not present in the closely related *Neosartorya fischeri *FRR0181. This sequence is located immediately after the conserved sequence block B of the splicing domain (Figure 3). It does not show significant sequence or structural similarity to other protein sequences in the databases. The intein in *B. cinerea *(Order: Helotiales) also contains a substantial insertion in this region (Figure 3). The sequence of the insertion in *Botrytis *shows 58% similarity to the insertion in the *A. fumigatus *inteins, although it is 33 residues longer. Other, different, indels occur in this region of the other ascomycete inteins and mini-inteins (Figure 3). It may be that this region is especially tolerant of substantial indels. After this variable region, but prior to the first motif of the homing endonuclease (block C), there is a region (~40 residues) that is relatively well conserved across all of the PRP8 inteins (including the mini-inteins) and which must presumably be part of the splicing domain. This region does not, however, show similarity to the N4 splicing domain motif recognised by Pietrokovski [35].

The splicing domains of the PRP8 inteins (Figure 1, In Base motifs A, B, F, G), whether from full-length or mini-inteins, are very closely similar to each other. The splicing domains from the ascomycete PRP8 inteins share more residues with those from the basidiomycete (*Cryptococcus*) PRP8 inteins than they do with the splicing domains of the saccharomycete (ascomycete) VMA inteins (Figure 1).

The internal regions of some of the PRP8 inteins evidently form part of an endonuclease due to their similarity to other homing endonuclease domains such as those in the VMA inteins. The homing endonuclease domains of the PRP8 inteins (Figure 1C, InBase motifs C, D, E, H) are closely similar to each other, although the spacing between the first two highly conserved motifs (C and D) is somewhat variable; for example, the distance between motifs C and D in the PRP8 full-length inteins ranges from 212 to 300 residues. Length variation in this region is also seen in the yeast VMA inteins; while in VMA inteins with active homing endonucleases this distance is ~80 residues, in inactive VMA homing endonucleases there may be as many as ~160 residues.

Many of the ascomycete PRP8 inteins found so far, are mini-inteins apparently without the homing endonuclease domain. The whole of the homing endonuclease domain is absent from all the ascomycete mini-inteins (as in the *Cryptococcus *mini-inteins). All of the mini-inteins are of approximately the same length and have no regions with vestiges of homing endonuclease motifs. The alignment of the mini-inteins with the homing endonuclease containing inteins (Figure 3) indicates that the homing endonuclease deletions have occurred at almost the identical site within the ascomycete mini-inteins. The *Cryptococcus *mini-inteins have an internal deletion of a similar length that also covers all the conserved homing endonuclease domains (Figure 3). The presence of numerous mini-inteins in the PRP8 allelic series contrasts with the VMA allelic series of inteins in saccharomycetes, all of which are full-length.

### Nucleotide changes and dS/dN values of PRP8 intein encoding sequences

The endonucleases of the yeast VMA intein series are frequently non-functional. It is of interest to determine if the newly described PRP8 HEG sequences encode active or inactive homing endonucleases. One way of approaching this question is to compare pairs of sequences and determine the frequencies of synonymous and non-synonymous changes in the HEGs of the inteins [36]. A large value for dS/dN implies that the encoded peptide is selectively constrained (functional). It should be borne in mind that since *PRP8 *is an essential gene, even if the homing endonuclease becomes non-functional, the intein must remain as an ORF and must encode functional splicing domains. The HEG domain is therefore still subject to weak selection pressure, even if it is inactive (for example stop codons and frame-shifts will be selected against).

We analysed the distribution of point mutations in the codons over a concatenated 213bp region of the HEG domains; this region encodes the nine residues of motif C and the whole region covering motifs D, E and H of the homing endonuclease (that is, the residues illustrated in Figure 1C). Results from analysis of the nine full-length PRP8 inteins are summarised in Table 2. The rate ratio of synonymous to non-synonymous substitutions (dS/dN) [37] was calculated via the syn-SCAN website [38,39]. The synonymous to non-synonymous rate provides a sensitive measure of selective pressure on the protein; a (dS/dN) value greater than 1.0 indicates that selection is operating to minimize the number of amino acid changes and thus retain the activity of the protein.

**Table 2:** Data describing nucleotide substitution patterns within the conserved regions of the homing endonuclease domains of pairs of full-length PRP8 inteins. Sd is the number of observed synonymous substitutions; Nd is the number of observed non-synonymous substitutions; S is the number of potential synonymous substitutions; N is the number of potential non synonymous substitutions; pS is the proportion of observed synonymous substitutions; pN is the proportion of observed non-synonymous substitutions; dS is the Jukes-Cantor correction for multiple hits of pS; dN is the Jukes-Cantor correction for multiple hits of pN. Inteins are identified using strain names as shown in Table 1.

**Seq1**	**Seq2**	**Sd**	**Nd**	**S**	**N**	**pS**	**pN**	**dS**	**dN**	**dS/dN**
Hca186AR	Hca217B	2.00	1.00	47.17	165.83	0.04	0.01	0.04	0.01	7.21
Hca186AR	BciPRP8	31.62	40.38	47.17	165.83	0.67	0.24	1.68	0.29	5.72
Hca186AR	PbrPRP8	36.00	32.00	48.67	164.33	0.74	0.19	3.22	0.23	14.27
Hca186AR	AniPRP8	41.88	38.12	46.50	166.50	0.90	0.23	NA	0.27	NA
Hca186AR	Afu0163	38.12	35.88	48.67	164.33	0.78	0.22	NA	0.26	NA
Hca186AR	Afu5109	38.12	36.88	48.33	164.67	0.79	0.22	NA	0.27	NA
Hca186AR	Nfi0181	36.38	33.62	48.33	164.67	0.75	0.20	NA	0.24	NA
Hca186AR	ClaPRP8	39.75	62.25	48.67	161.33	0.82	0.39	NA	0.54	NA
										
Hca217B	BciPRP8	31.12	39.88	47.67	165.33	0.65	0.24	1.53	0.29	5.27
Hca217B	PbrPRP8	35.50	31.50	49.17	163.83	0.72	0.19	2.47	0.22	11.10
Hca217B	AniPRP8	42.75	37.25	47.00	166.00	0.91	0.22	NA	0.27	NA
Hca217B	Afu0163	37.62	35.38	49.17	163.83	0.77	0.22	NA	0.25	NA
Hca217B	Afu5109	37.62	36.38	48.83	164.17	0.77	0.22	NA	0.26	NA
Hca217B	Nfi0181	35.88	33.12	48.83	164.17	0.73	0.20	2.92	0.24	12.41
Hca217B	ClaPRP8	39.00	60.00	49.17	160.83	0.79	0.37	NA	0.52	NA
										
BciPRP8	PbrPRP8	43.50	27.50	49.17	163.83	0.88	0.17	NA	0.19	NA
BciPRP8	AniPRP8	39.00	24.00	47.00	166.00	0.83	0.14	NA	0.16	NA
BciPRP8	Afu0163	39.50	16.50	49.17	163.83	0.80	0.10	NA	0.11	NA
BciPRP8	Afu5109	39.50	17.50	48.83	164.17	0.81	0.11	NA	0.11	NA
BciPRP8	Nfi0181	35.50	16.50	48.83	164.17	0.73	0.10	2.61	0.11	24.21
BciPRP8	ClaPRP8	36.75	46.25	49.50	160.50	0.74	0.29	3.45	0.36	9.48
										
PbrPRP8	AniPRP8	38.88	31.12	48.50	164.50	0.80	0.19	NA	0.22	NA
PbrPRP8	Afu0163	38.25	23.75	50.67	162.33	0.75	0.15	NA	0.16	NA
PbrPRP8	Afu5109	38.25	24.75	50.33	162.67	0.76	0.15	NA	0.17	NA
PbrPRP8	Nfi0181	40.25	22.75	50.33	162.67	0.80	0.14	NA	0.15	NA
PbrPRP8	ClaPRP8	38.88	48.12	51.00	159.00	0.76	0.30	NA	0.39	NA
										
AniPRP8	Afu0163	36.62	22.38	48.50	164.50	0.76	0.14	NA	0.15	NA
AniPRP8	Afu5109	35.62	22.38	48.17	164.83	0.74	0.14	3.21	0.15	21.44
AniPRP8	Nfi0181	38.62	20.38	48.17	164.83	0.80	0.12	NA	0.14	NA
AniPRP8	ClaPRP8	44.50	43.50	48.83	161.17	0.91	0.27	NA	0.33	NA
										
Afu0163	Afu5109	1.00	0.00	50.33	162.67	0.02	0.00	0.02	0.00	NA
Afu0163	Nfi0181	11.00	2.00	50.33	162.67	0.22	0.01	0.26	0.01	20.84
Afu0163	ClaPRP8	40.50	47.50	51.00	159.00	0.79	0.30	NA	0.38	NA
										
Afu5109	Nfi0181	12.00	2.00	50.00	163.00	0.24	0.01	0.29	0.01	23.38
Afu5109	ClaPRP8	41.50	47.50	50.67	159.33	0.82	0.30	NA	0.38	NA
										
Nfi0181	ClaPRP8	39.62	47.38	50.67	159.33	0.78	0.30	NA	0.38	NA

Comparison of the HEG regions of closely related fungi gave high dS/dN ratios. For example comparison of the HEG region of the *A. fumigatus *intein with the HEG region of its closest relative, *N. fischeri*, yields a dS/dN value of 20.84, indicating that the rate of synonymous (neutral) change far outweighs the rate of non-synonymous change. It is therefore likely that selection is acting to reduce change in these sequences by eliminating mutant alleles carrying non-synonymous substitutions. This indicates that the HEG region of these inteins encodes active endonucleases (or did so until recently). Comparisons between *Histoplasma *and *Paracoccidioides *gave a dS/dN of 14.27. Even some less closely related pairs gave high dS/dN values, for example the *Aspergillus nidulans*/*Aspergillus fumigatus *dS/dN is 21.44 and the *Botrytis cinerea*/*Neosartorya fischeri *dS/dN is 24.21. The calculation of the dS/dN value was not possible for many pairs because the frequency of synonymous substitutions was greater than 0.74, which makes calculating the Jukes-Cantor correction unreliable (that is, the system is saturated by synonymous substitutions in highly diverged sequences). Several comparisons, especially those between the more distantly related species (for example all comparisons involving *Cryptococcus laurentii)*, encountered this limitation. These dS/dN values are entered as NA in Table 2. However, consideration of the underlying substitution frequencies shows clearly that even these comparisons are reflecting strong selective pressure. For example, in Table 2, the comparison of *Histoplasma *(Hca217B) with Nfi0181, allows a dS/dN value to be calculated, the comparison gives a pS of 0.73, a pN of 0.2 and a dS/dN of 12.41. In contrast, comparison of the other *Histoplasma *intein (Hca186AR) with *Neosartorya fischeri *(Nfi0181) does not allow a dS/dN value to be calculated; it gives a pS of 0.75 (which only just exceeds the Jukes-Cantor limit, 0.74) a pN of 0.2 and therefore the dS/dN value cannot be calculated. Many of the comparisons listed as NA in Table 2 are only just beyond calculation (due to the Jukes-Cantor limit) and are very similar to comparisons that give clear evidence of selective constraint (high dS/dN values).

In conclusion, dS/dN analysis of the PRP8 HEGs suggests that they are constrained by selection. In addition, the high frequency of synonymous substitutions indicates that these inteins have been diverging for a considerable period of time.

### Nucleotide changes and dS/dN values of VMA intein encoding sequences

It is of interest to compare the nucleotide substitution pattern in the PRP8 HEGs (described above) with a similar analysis of the yeast VMA HEGs. It has been shown that some of these VMA homing endonucleases are inactive [9]. The longer a homing endonuclease has been inactive, the more random the pattern of substitutions will be (dS/dN will approach 1.0). We have analysed the substitution patterns between VMA HEGs from 17 strains from 16 yeast species, using the same HEG region used to analyse the PRP8 homing endonucleases (Table 3). Of these 17, four have been shown to have active homing endonucleases (*S. cerevisiae*, *S. cerevisiae *DH1-1A,*S. cariocanus*, *Zygosaccharomyces baillii*), two homing endonucleases have not had their activity determined experimentally (*Candida glabrata *and *Debaryomyces hansenii*) and the remaining 11 homing endonucleases are inactive [9].

**Table 3:** Data describing nucleotide substitution patterns within the conserved regions of the homing endonuclease domains of pairs of VMA inteins. Sd is the number of observed synonymous substitutions; Nd is the number of observed non-synonymous substitutions; S is the number of potential synonymous substitutions; N is the number of potential non-synonymous substitutions; pS is the proportion of observed synonymous substitutions; pN is the proportion of observed non-synonymous substitutions; dS is the Jukes-Cantor correction for multiple hits of pS; dN is the Jukes-Cantor correction for multiple hits of pN. EN describes the experimentally determined activity of the homing endonuclease [9], A= active, I= inactive. The activities of the endonucleases from C. glabrata and D. hansenii have not been determined, but are likely to be inactive. The species in which VMA inteins occur are labelled: Scerev, Saccharomyces cerevisiae; SspDH1A, a wild isolate of S. cerevisiae; Scarioc, S. cariocanus; Tglobosa, Torulaspora globosa; Zbailli, Zygosaccharomyces bailli; Tpret, T. pretoriensis; Zbisporus, Zygosaccharomyces bisporus; Zrouxii, Z. rouxii; Kpolysp, Kluyveromyces polysporus; Sunisp, S. (Kazachstania) unisporus; Sexiguus, S. (Kazachstania) exiguus; Ctropicalis, Candida tropicalis; Sdair, S. (Naumovia) dairenensis; Scastelli, S. (Naumovia) castellii; Cglabrata, C. glabrata; Klactis, K. lactis; Dhansenii, Debaryomyces hansenii. Inteins with active homing endonucleases are marked by asterisks*.

		Sd	Nd	S	N	pS	pN	dS	dN	dS/dN	EN
*Scerev	*SspDH1A	9.00	0.00	52.00	158.00	0.17	0.00	0.20	0.00	NA	AA
*Scerev	*Scarioc	13.00	4.00	52.33	157.67	0.25	0.03	0.30	0.03	11.69	AA
*Scerev	Tglobosa	31.88	36.12	52.50	157.50	0.61	0.23	1.24	0.27	4.54	AI
*Scerev	*Zbailli	31.50	34.50	51.50	158.50	0.61	0.22	1.27	0.26	4.93	AA
*Scerev	Tpret	34.62	41.38	52.83	157.17	0.66	0.26	1.55	0.32	4.79	AI
*Scerev	Zbisporus	27.00	38.00	49.33	151.67	0.55	0.25	0.98	0.30	3.22	AI
*Scerev	Zrouxii	22.38	41.62	48.50	152.50	0.46	0.27	0.72	0.34	2.11	AI
*Scerev	Kpolysp	30.88	57.12	48.00	162.00	0.64	0.35	1.46	0.48	3.07	AI
*Scerev	Sdair	34.75	61.25	51.50	158.50	0.67	0.39	1.72	0.54	3.18	AI
*Scerev	Sunisp	27.62	70.38	52.17	157.83	0.53	0.45	0.92	0.68	1.36	AI
*Scerev	Sexiguus	24.00	71.00	50.67	159.33	0.47	0.45	0.75	0.68	1.11	AI
*Scerev	Ctropicalis	28.75	70.25	52.17	157.83	0.55	0.45	1.00	0.68	1.47	AI
*Scerev	Scastelli	35.88	56.12	51.00	159.00	0.70	0.35	2.08	0.48	4.37	AI
*Scerev	Cglabrata	25.38	67.62	45.83	143.17	0.55	0.47	1.01	0.75	1.35	AI
*Scerev	Klactis	33.12	70.88	49.50	151.50	0.67	0.47	1.67	0.73	2.28	AI
*Scerev	Dhansenii	32.62	50.38	52.83	154.17	0.62	0.33	1.30	0.43	3.03	AI
*SspDH1A	*Scarioc	15.00	4.00	52.33	157.67	0.29	0.03	0.36	0.03	13.99	AA
*SspDH1A	Tglobosa	28.12	35.88	52.50	157.50	0.54	0.23	0.94	0.27	3.46	AI
*SspDH1A	*Zbailli	29.25	34.75	51.50	158.50	0.57	0.22	1.06	0.26	4.09	AA
*SspDH1A	Tpret	35.38	41.62	52.83	157.17	0.67	0.26	1.67	0.33	5.13	AI
*SspDH1A	Zbisporus	23.75	38.25	49.33	151.67	0.48	0.25	0.77	0.31	2.51	AI
*SspDH1A	Zrouxii	23.62	41.38	48.50	152.50	0.49	0.27	0.79	0.34	2.33	AI
*SspDH1A	Kpolysp	30.38	56.62	48.00	162.00	0.63	0.35	1.39	0.47	2.96	AI
*SspDH1A	Sdair	33.50	61.50	51.50	158.50	0.65	0.39	1.51	0.55	2.77	AI
*SspDH1A	Sunisp	26.88	71.12	52.17	157.83	0.52	0.45	0.87	0.69	1.26	AI
*SspDH1A	Sexiguus	23.75	71.25	50.67	159.33	0.47	0.45	0.74	0.68	1.08	AI
*SspDH1A	Ctropicalis	27.00	70.00	52.17	157.83	0.52	0.44	0.88	0.67	1.31	AI
*SspDH1A	Scastelli	34.62	56.38	51.00	159.00	0.68	0.35	1.77	0.48	3.68	AI
*SspDH1A	Cglabrata	27.12	67.88	45.83	143.17	0.59	0.47	1.17	0.75	1.56	AI
*SspDH1A	Klactis	33.88	71.12	49.50	151.50	0.68	0.47	1.83	0.74	2.48	AI
*SspDH1A	Dhansenii	32.62	50.38	52.83	154.17	0.62	0.33	1.30	0.43	3.03	AI
*Scarioc	Tglobosa	31.50	35.50	52.83	157.17	0.60	0.23	1.19	0.27	4.42	AI
*Scarioc	*Zbailli	32.88	33.12	51.83	158.17	0.63	0.21	1.40	0.25	5.71	AA
*Scarioc	Tpret	38.12	41.88	53.17	156.83	0.72	0.27	2.34	0.33	7.10	AI
*Scarioc	Zbisporus	27.50	37.50	49.33	151.67	0.56	0.25	1.02	0.30	3.40	AI
*Scarioc	Zrouxii	28.12	38.88	48.50	152.50	0.58	0.25	1.11	0.31	3.57	AI
*Scarioc	Kpolysp	27.88	59.12	48.33	161.67	0.58	0.37	1.10	0.50	2.19	AI
*Scarioc	Sdair	34.62	63.38	51.83	158.17	0.67	0.40	1.66	0.57	2.90	AI
*Scarioc	Sunisp	31.12	69.88	52.50	157.50	0.59	0.44	1.17	0.67	1.75	AI
*Scarioc	Sexiguus	25.75	72.25	51.00	159.00	0.50	0.45	0.84	0.70	1.20	AI
*Scarioc	Ctropicalis	27.38	68.62	52.50	157.50	0.52	0.44	0.89	0.65	1.37	AI
*Scarioc	Scastelli	38.88	57.12	51.33	158.67	0.76	0.36	NA	0.49	NA	AI
*Scarioc	Cglabrata	23.50	69.50	45.83	143.17	0.51	0.49	0.86	0.78	1.10	AI
*Scarioc	Klactis	35.12	72.88	49.50	151.50	0.71	0.48	2.19	0.77	2.85	AI
*Scarioc	Dhansenii	29.75	49.25	52.83	154.17	0.56	0.32	1.04	0.42	2.50	AI
Tglobosa	*Zbailli	24.50	13.50	52.00	158.00	0.47	0.09	0.74	0.09	8.18	IA
Tglobosa	Tpret	28.50	19.50	53.33	156.67	0.53	0.12	0.93	0.14	6.87	II
Tglobosa	Zbisporus	26.12	35.88	49.33	151.67	0.53	0.24	0.92	0.28	3.23	II
Tglobosa	Zrouxii	22.50	31.50	48.50	152.50	0.46	0.21	0.72	0.24	2.99	II
Tglobosa	Kpolysp	24.50	65.50	48.50	161.50	0.51	0.41	0.84	0.58	1.44	II
Tglobosa	Sdair	32.00	64.00	52.00	158.00	0.62	0.41	1.29	0.58	2.21	II
Tglobosa	Sunisp	29.50	65.50	52.67	157.33	0.56	0.42	1.03	0.61	1.70	II
Tglobosa	Sexiguus	28.12	73.88	51.17	158.83	0.55	0.47	0.99	0.73	1.36	II
Tglobosa	Ctropicalis	32.25	72.75	52.67	157.33	0.61	0.46	1.27	0.72	1.77	II
Tglobosa	Scastelli	35.50	52.50	51.50	158.50	0.69	0.33	1.89	0.44	4.31	II
Tglobosa	Cglabrata	26.00	82.00	45.50	143.50	0.57	0.57	1.08	1.08	1.00	II
Tglobosa	Klactis	33.75	73.25	49.50	151.50	0.68	0.48	1.80	0.78	2.32	II
Tglobosa	Dhansenii	31.25	55.75	53.00	154.00	0.59	0.36	1.16	0.49	2.34	II
*Zbailli	Tpret	32.75	21.25	52.33	157.67	0.63	0.13	1.35	0.15	9.08	AI
*Zbailli	Zbisporus	30.62	33.38	48.50	152.50	0.63	0.22	1.38	0.26	5.35	AI
*Zbailli	Zrouxii	31.75	31.25	47.67	153.33	0.67	0.20	1.64	0.24	6.91	AI
*Zbailli	Kpolysp	30.75	65.25	47.50	162.50	0.65	0.40	1.49	0.57	2.59	AI
*Zbailli	Sdair	31.62	63.38	51.00	159.00	0.62	0.40	1.31	0.57	2.31	AI
*Zbailli	Sunisp	30.38	68.62	51.67	158.33	0.59	0.43	1.15	0.65	1.78	AI
*Zbailli	Sexiguus	29.00	73.00	50.17	159.83	0.58	0.46	1.10	0.70	1.57	AI
*Zbailli	Ctropicalis	35.75	74.25	51.67	158.33	0.69	0.47	1.92	0.74	2.61	AI
*Zbailli	Scastelli	31.25	53.75	50.50	159.50	0.62	0.34	1.31	0.45	2.92	AI
*Zbailli	Cglabrata	21.62	76.38	44.67	144.33	0.48	0.53	0.78	0.92	0.85	AI
*Zbailli	Klactis	28.88	71.12	48.67	152.33	0.59	0.47	1.17	0.73	1.61	AI
*Zbailli	Dhansenii	34.75	59.25	52.00	155.00	0.67	0.38	1.66	0.53	3.11	AI
Tpret	Zbisporus	28.38	36.62	50.00	151.00	0.57	0.24	1.06	0.29	3.62	II
Tpret	Zrouxii	26.00	37.00	49.17	151.83	0.53	0.24	0.92	0.29	3.11	II
Tpret	Kpolysp	27.38	59.62	48.83	161.17	0.56	0.37	1.03	0.51	2.02	II
Tpret	Sdair	27.62	62.38	52.33	157.67	0.53	0.40	0.91	0.56	1.62	II
Tpret	Sunisp	27.62	71.38	53.00	157.00	0.52	0.45	0.89	0.70	1.27	II
Tpret	Sexiguus	32.00	71.00	51.50	158.50	0.62	0.45	1.32	0.68	1.94	II
Tpret	Ctropicalis	30.62	65.38	53.00	157.00	0.58	0.42	1.10	0.61	1.82	II
Tpret	Scastelli	29.00	55.00	51.83	158.17	0.56	0.35	1.03	0.47	2.20	II
Tpret	Cglabrata	29.88	80.12	46.50	142.50	0.64	0.56	1.46	1.04	1.40	II
Tpret	Klactis	38.38	77.62	50.17	150.83	0.76	0.51	NA	0.87	NA	II
Tpret	Dhansenii	34.25	57.75	53.33	153.67	0.64	0.38	1.45	0.52	2.79	II
Zbisporus	Zrouxii	18.12	25.88	47.83	153.17	0.38	0.17	0.53	0.19	2.76	II
Zbisporus	Kpolysp	27.38	55.62	45.67	155.33	0.60	0.36	1.20	0.49	2.47	II
Zbisporus	Sdair	29.25	59.75	48.67	152.33	0.60	0.39	1.21	0.56	2.18	II
Zbisporus	Sunisp	27.38	61.62	49.00	152.00	0.56	0.41	1.02	0.58	1.76	II
Zbisporus	Sexiguus	19.25	62.75	48.50	152.50	0.40	0.41	0.57	0.60	0.95	II
Zbisporus	Ctropicalis	26.75	66.25	49.33	151.67	0.54	0.44	0.96	0.65	1.47	II
Zbisporus	Scastelli	30.50	55.50	48.33	152.67	0.63	0.36	1.38	0.50	2.78	II
Zbisporus	Cglabrata	24.88	71.12	44.50	144.50	0.56	0.49	1.03	0.80	1.28	II
Zbisporus	Klactis	33.38	76.62	48.83	152.17	0.68	0.50	1.82	0.83	2.18	II
Zbisporus	Dhansenii	29.88	58.12	50.33	150.67	0.59	0.39	1.18	0.54	2.17	II
Zrouxii	Kpolysp	24.00	58.00	44.83	156.17	0.54	0.37	0.94	0.51	1.83	II
Zrouxii	Sdair	28.50	63.50	47.83	153.17	0.60	0.41	1.19	0.60	1.97	II
Zrouxii	Sunisp	27.88	68.12	48.17	152.83	0.58	0.45	1.11	0.68	1.64	II
Zrouxii	Sexiguus	24.50	64.50	47.67	153.33	0.51	0.42	0.87	0.62	1.40	II
Zrouxii	Ctropicalis	25.88	66.12	48.50	152.50	0.53	0.43	0.93	0.65	1.44	II
Zrouxii	Scastelli	24.50	56.50	47.50	153.50	0.52	0.37	0.87	0.51	1.72	II
Zrouxii	Cglabrata	29.38	79.62	44.33	144.67	0.66	0.55	1.61	0.99	1.62	II
Zrouxii	Klactis	31.38	76.62	48.00	153.00	0.65	0.50	1.54	0.83	1.86	II
Zrouxii	Dhansenii	29.75	55.25	49.50	151.50	0.60	0.36	1.21	0.50	2.43	II
Kpolysp	Sdair	19.25	69.75	48.33	164.67	0.40	0.42	0.57	0.62	0.91	II
Kpolysp	Sunisp	19.88	77.12	49.17	163.83	0.40	0.47	0.58	0.74	0.78	II
Kpolysp	Sexiguus	21.12	74.88	47.67	165.33	0.44	0.45	0.67	0.69	0.97	II
Kpolysp	Ctropicalis	20.25	78.75	49.00	164.00	0.41	0.48	0.60	0.77	0.78	II
Kpolysp	Scastelli	28.50	71.50	47.67	165.33	0.60	0.43	1.20	0.64	1.86	II
Kpolysp	Cglabrata	20.12	70.88	42.50	146.50	0.47	0.48	0.75	0.78	0.96	II
Kpolysp	Klactis	28.38	80.62	45.83	155.17	0.62	0.52	1.31	0.89	1.48	II
Kpolysp	Dhansenii	30.12	70.88	48.67	158.33	0.62	0.45	1.31	0.68	1.92	II
Sdair	Sunisp	19.25	48.75	53.50	165.50	0.36	0.29	0.49	0.37	1.31	II
Sdair	Sexiguus	21.62	59.38	52.00	167.00	0.42	0.36	0.61	0.48	1.26	II
Sdair	Ctropicalis	26.12	48.88	52.67	163.33	0.50	0.30	0.81	0.38	2.13	II
Sdair	Scastelli	30.38	25.62	51.00	162.00	0.60	0.16	1.19	0.18	6.67	II
Sdair	Cglabrata	23.75	76.25	44.67	144.33	0.53	0.53	0.93	0.91	1.01	II
Sdair	Klactis	29.25	75.75	48.83	152.17	0.60	0.50	1.20	0.82	1.47	II
Sdair	Dhansenii	30.38	55.62	52.00	155.00	0.58	0.36	1.13	0.49	2.32	II
Sunisp	Sexiguus	17.12	61.88	53.17	165.83	0.32	0.37	0.42	0.52	0.82	II
Sunisp	Ctropicalis	21.50	59.50	53.50	162.50	0.40	0.37	0.58	0.50	1.15	II
Sunisp	Scastelli	29.88	57.12	51.83	161.17	0.58	0.35	1.10	0.48	2.29	II
Sunisp	Cglabrata	23.00	65.00	45.00	144.00	0.51	0.45	0.86	0.69	1.24	II
Sunisp	Klactis	27.88	67.12	49.17	151.83	0.57	0.44	1.06	0.67	1.58	II
Sunisp	Dhansenii	28.50	69.50	52.67	154.33	0.54	0.45	0.96	0.69	1.39	II
Sexiguus	Ctropicalis	21.25	65.75	52.33	163.67	0.41	0.40	0.58	0.58	1.02	II
Sexiguus	Scastelli	35.00	55.00	50.33	162.67	0.70	0.34	1.96	0.45	4.37	II
Sexiguus	Cglabrata	17.88	73.12	44.83	144.17	0.40	0.51	0.57	0.85	0.67	II
Sexiguus	Klactis	29.12	81.88	48.67	152.33	0.60	0.54	1.20	0.95	1.27	II
Sexiguus	Dhansenii	25.50	65.50	51.50	155.50	0.50	0.42	0.81	0.62	1.31	II
Ctropicalis	Scastelli	36.25	55.75	51.67	161.33	0.70	0.35	2.06	0.46	4.44	II
Ctropicalis	Cglabrata	29.38	74.62	45.50	143.50	0.65	0.52	1.48	0.89	1.67	II
Ctropicalis	Klactis	32.25	78.75	49.50	151.50	0.65	0.52	1.52	0.89	1.72	II
Ctropicalis	Dhansenii	32.00	66.00	52.67	154.33	0.61	0.43	1.25	0.63	1.97	II
Scastelli	Cglabrata	27.88	83.12	44.83	144.17	0.62	0.58	1.32	1.10	1.21	II
Scastelli	Klactis	31.50	78.50	48.50	152.50	0.65	0.51	1.51	0.87	1.73	II
Scastelli	Dhansenii	33.38	53.62	51.83	155.17	0.64	0.35	1.47	0.46	3.17	II
Cglabrata	Klactis	26.38	61.62	44.83	144.17	0.59	0.43	1.15	0.63	1.82	II
Cglabrata	Dhansenii	22.75	81.25	46.50	142.50	0.49	0.57	0.79	1.07	0.74	II
Klactis	Dhansenii	34.12	71.88	50.50	150.50	0.68	0.48	1.73	0.76	2.28	II

Comparison of substitution patterns of active VMA homing endonucleases gave dS/dN values ranging from 4.09 (*Z. baillii*/*S. cerevisiae *DH1-1A) to 13.99 (*S. cerevisiae *DH11-1A */S. cariocanus*). Comparison of substitution patterns of inactive VMA homing endonucleases gave dS/dN values from 0.67 (*S. exiguus*/*Candida glabrata*) to 6.87 (*Torulaspora pretoriensis*/*T. globosa*). The VMA homing endonuclease from these two species of *Torulaspora *is closely similar to that of *Z. baillii*, a homing endonuclease known to be functional. It is likely that the *Torulaspora *homing endonucleases have only recently become inactive (resulting in a residual high dS/dN value). Of the 74 comparisons between inactive homing endonucleases, 70 give dS/dN values below 4. Of the 52 comparisons with mixed pairs (active homing endonucleases compared with inactive ones), there were only 11 instances where the dS/dN value was >4.0. Nine of these involved endonucleases from species of *Torulaspora *that are probably only recently inactive.

In summary, the correlation of the dS/dN values and homing endonuclease activity in the VMA cohort supports the concept of using dS/dN analyses to predict the activity of the PRP8 intein homing endonucleases. In other words, the comparison of active homing endonucleases gave high dS/dN values; in contrast comparison of inactive homing endonucleases gave low dS/dN values. The high dS/dN values of the PRP8 homing endonucleases are strikingly greater than the VMA active homing endonuclease values. Also, the frequency of synonymous substitutions was less than 0.74 in all but one VMA comparison, that is, the VMA system is not saturated by synonymous substitutions. Taken at face value this implies that the saccharomycete VMA intein allelic group is of more recent origin than the PRP8 allelic group.

### Comparison of the nucleotide changes of the VMA and PRP8 intein encoding sequences

The above dS/dN analyses showed numerous comparisons where the Jukes-Cantor correction could not be calculated because of the high frequency of synonymous substitutions. These comparisons are described as NA in Table 2. In order to examine the homing endonuclease evolution in a way that includes all the comparisons, we submitted our data to analysis at the SNAP (Synonymous/Non-synonymous Analysis Program) site [40]. SNAP calculates rates of nucleotide substitution from a set of codon-aligned nucleotide sequences, based on the method of Nei and Gojobori [37]. The XY-Plot function at this site provides an illustration of the cumulative behaviour of the synonymous and non-synonymous substitutions across the coding region, one codon at a time; that is, the average behaviour at each codon is estimated from all the pair-wise comparisons. In the PRP8 HEG region, the synonymous and non-synonymous substitutions accumulate at a similar rate (despite there being many more non-synonymous options per codon). In the VMA HEG, non-synonymous substitutions accumulate at almost twice the rate as those in the PRP8 HEG region (see Additional file 1: SNAP XY plots of the homing endonuclease encoding regions of the PRP8 and VMA inteins). This analysis, which is based on the complete data set, supports the individual dS/dN comparisons.

### PRP8 and VMA intein substitutions at the Asp active sites

To explore the activity of the PRP8 homing endonucleases further we aligned the active site residues [9,36] of the homing endonucleases from PRP8 and yeast VMA inteins. As an example, the two aspartic acid residues (Asp-218 and Asp-326 in the *S. cerevisiae *VMA intein; marked by asterisks in Figure 1C) are involved in the active site co-ordination of a divalent metal ion, and are critical for activity of the homing endonuclease. These two aspartic acid residues are conserved in the active VMA homing endonucleases (*S. cerevisiae*, *S. cariocanus *and *Z. baillii*). The only inactive homing endonucleases to retain both these sites are from the two *Torulaspora *species; these homing endonucleases are closely similar to the active *Z. baillii *homing endonuclease. We believe, therefore, that the *Torulaspora *homing endonucleases have only recently become inactive. Of the two VMA homing endonucleases whose functionality is unknown, the one from *C. glabrata *lacks both these critical aspartic acid residues and the *D. hansenii *homing endonuclease lacks the proximal aspartic acid. The homing endonuclease domains of all of the ascomycete PRP8 inteins, except PbrPRP8 and ClaPRP8, have both these critical aspartates, supporting the belief that these homing endonucleases are active.

### Euascomycete fungi lacking a PRP8 intein

During our systematic search for further PRP8 inteins using the public sequence databases (last search September 26th 2005), we encountered several instances where there was no PRP8 intein in close relatives of species known to contain an intein (see Table 4 for the complete list). For example, *Coccidiodes posadasii *and *Coccidiodes immitis *(Order: Onygales) are very closely related to *Uncinocarpus reesii *(Bowman et al., 1996), but neither species of *Coccidioides *contains an intein, whereas *U. reesii *does (Table 1, Table 4). In the family Sclerotiniaceae, *B. cinerea *and *Sclerotinia sclerotiorum *are very closely related [42] but there is no PRP8 intein in *S. sclerotiorum *(Table 4). Several species of *Aspergillus *are also known not to have a PRP8 intein [25] and (Table 4). Within the section Clavati of the genus *Aspergillus *there are two major clusters of species [29,31]. One cluster includes *A. clavatus *(which has no PRP8 intein) and the other includes *A. giganteus*, which has a PRP8 mini-intein quite distinct from those present in species from the section Fumigati. Within the Fumigati, only one of the species analysed (*A. unilateralis*) was without an intein.

**Table 4:** Fungal species without an intein in PRP8. Detected by a survey of PRP8 intein insertion sites of species in public databases using TBLASTN with a partial, intein-less, sequence of the *Aspergillus fumigatus *PRP8 as the query. The presence/absence of an intron almost immediately downstream of the empty intein insertion site is also noted. Included are species shown not to have an intein by Liu & Yang [25] and Butler & Poulter [20]. Note that the data represent only one isolate from each species.

Species	Order	Accession	intron
	ASCOMYCOTA		

*Schizosaccharomyces pombe*	Schizosaccharomycetales	NC_003424.1	no
*Pneumocystis carinii*	Pneumocystidales	contig_130	no
			

	Ascomycotina		

*Pichia guilliermondii*	Saccharomycetales	AAFM01000018	no
*Saccharomyces kluyveri*	"	AACE01000391.1	no
*Candida tropicalis*	"	AAFN01000124.1	no
*Candida albicans*	"	AACQ01000072.1	no
*Candida glabrata*	"	NC_006032.1	no
*Saccharomyces paradoxus*	"	AABY01000045.1	no
*Saccharomyces cerevisiae*	"	NC_001140.4	no
*Saccharomyces bayanus*	"	AACA01000109.1	no
*Saccharomyces mikatae*	"	AABZ01000010.1	no
*Kluyveromyces waltii*	"	AADM01000296.1	no
*Kluyveromyces lactis*	"	NC_006037.1	no
*Eremothecium gossypii*	"	NC_005783.2	no
*Debaryomyces hansenii*	"	NC_006047.1	no
*Yarrowia lipolytica*	"	NC_006069.1	no
*Clavispora lusitaniae*	"	AAFT01000064	no
			

	Pezizomycotina		

*Aspergillus terreus*	Eurotiales	AAJN01000238.1	no
*Aspergillus clavatus*	"	AAKD01000005.1	yes
*Aspergillus flavus*	"	AAIH01001496.1	no
*Aspergillus niger*	"	Liu & Yang 2004	?
*Aspergillus oryzae*	"	Liu & Yang 2004	?
*Aspergillus parasiticus*	"	Liu & Yang 2004	?
*Aspergillus ustus*	"	Liu & Yang 2004	?
*Aspergillus lentulus*	"	This work	yes
*Phaeosphaeria nodorum*	Pleosporales	AAGI01000010	yes
*Neurospora crassa*	Sordariales	AABX01000169	no
*Chaetomium globosum*	Sordariales	AAFU01000976	yes
*Podospora anserina*	Sordariales	contig_159	yes
*Magnaporthe grisea*	Sordariales	AACU01000805	no
*Trichoderma reesei*	Hypocreales	AAIL01001072	no
*Gibberella zeae PH-1*	Hypocreales	AACM01000127	no
*Gibberella moniliformis*	Hypocreales	contig 2.11	no
*Coccidioides posadasii*	Onygenales	CF811886	no
*Coccidioides immitis*	Onygenales	AAEC01000088	no
*Sclerotinia sclerotiorum*	Helotiales	supercontig_1.4	yes
			

	BASIDIOMYCOTA		

*Phanerochaete chrysosporium*	Aphyllophorales	AADS01000435.1	no
*Coprinopsis cinerea*	Agaricales	AACS01000175	no
*Ustilago maydis*	Ustilaginales	AACP01000056.1	no
*Cryptococcus amylolentus*	Tremellales	Butler & Poulter 2005	yes
*Cryptococcus dimmenae*	"	Butler & Poulter 2005	yes
*Cryptococcus heveanensis*	"	Butler & Poulter 2005	yes
*Cryptococcus albidus*	"	Butler & Poulter 2005	no
*Cryptococcus uniguttulatus*	"	Butler & Poulter 2005	no
			

	ZYGOMYCOTA		

*Rhizopus oryzae*		supercontig_1.5	no

PRP8 inteins may eventually be found in other isolates of some of these apparently 'intein-less' species. The data in Table 4 are derived from single isolates of each species and it is possible that species will be found that are polymorphic for the PRP8 intein.

### Distribution of PRP8 inteins

**Figure 4:**
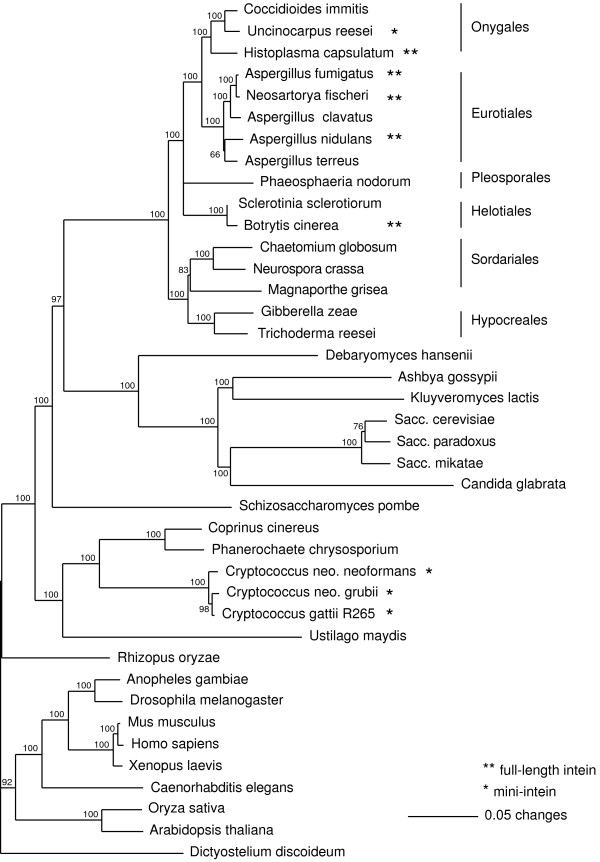
**Phylogenetic tree based on an alignment of PRP8 proteins**. The tree was constructed by the neighbour-joining method using PAUP* [61] and is a consensus derived from 1000 bootstrap replicates. The numbers indicate the percentage bootstrap support (only nodes with >50% support are shown). Accession numbers for the PRP8 sequences are: *Caenorhabditis elegans*, NM_066384; *Mus musculus*, AB047391; *Homo sapiens*, BAA22563; *Xenopus laevis*, AAH45266; *Anopheles gambiae*, EAA04255; *Drosophila melanogaster*, NP610735; *Oryza sativa*, AB023482; *Arabidopsis thaliana*, AC009322; *Schizosaccharomyces pombe*, T38841; *Neurospora crassa*, EAA33717; *Aspergillus nidulans*, BK001316; *Saccharomyces cerevisiae*, S34670; *S. paradoxus*, AABY01000045 (ORF: 35077-42312); *S. mikatae*, AABZ01000010 (ORF: 37550-44800); *Neurospora crassa *(EAA33717); *Dictyostelium discoideum*, AAL92617. PRP8 sequences of *Candida glabrata *(gene name CAGL0I02266g), *Debaryomyces hansenii *(gene name DEHA0E02717g) and *Kluyveromyces lactis *(gene name KLLA0A05280g) were obtained from the Génolevures website. Predicted PRP8 protein sequences for the remaining species were generated using data from their respective genome sequencing web sites and are available from the authors. Accession numbers refer to data which include the PRP8 encoding regions; *Cryptococcus neoformans *var. *grubii *(AACO01000004), *Cryptococcus neoformans *var.*neoformans *(AAEY01000001), *Cryptococcus gattii *R265 (AAFP01000516), *Coprinopsis cinerea *(AACS01000175), *Gibberella zeae *(AACM01000127), *Ashbya gossypii *(AAS50980), *Aspergillus fumigatus *(AAHF01000008), *Aspergillus clavatus *(AAKD01000005), *Aspergillus terreus *(AAJN01000238), *Neosartorya fischeri *(AAKE01000005.1), *Uncinocarpus reesii *(AAIW01000130.1), *Coccidioides immitis *(AAEC02000024), *Trichoderma reesei *(AAIL01001072), *Magnaporthe grisea *(AACU02000289), *Chaetomium globosum *(AAFU01000976), *Phaeosphaeria nodorum *(AAGI01000010), *Ustilago maydis *(AACP01000056), *Phanerochaete chrysosporium *(AADS01000435), *Botrytis cinerea *(AAID01001534, AAID01001533), *Sclerotinia sclerotiorum *(AAGT01000110), *Rhizopus oryzae *(AACW02000011), and *Histoplasma capsulatum *(AAJI01001309).

In addition to the sporadic nature of PRP8 intein distribution within the euascomycetes mentioned above, the wider distribution of the PRP8 intein is also decidedly sporadic (Figure 4) (Table 4). The intein is present in species from three orders of euascomycetes (Pezizomycotina) and a very narrow range of basidiomycete species. This would suggest a widespread occurrence and an ancient origin for the intein within the euascomycetes; however most euascomycetes for which there is PRP8 sequence data available do not have an intein in PRP8. There is no intein encoded in the *PRP8 *gene of any species from the other major ascomycete class, hemiascomycetes (Saccharomycotina); for example, *Candida albicans*, *Candida guilliermondii*, *Eremothecium gossypii, Kluyveromyces waltii*, *Saccharomyces cerevisiae*. Neither of the two archaeascomycetes (*Schizosaccharomyces pombe, Pneumocystis carinii*) for which PRP8 sequence data are available encodes a PRP8 intein. In summary, if the PRP8 intein is descended from a precursor present in the common ancestor of the Ascomycota, the loss of the intein must have been a frequent occurrence, either by deletion or by fixation of an empty allele.

The only other PRP8 inteins are present in four species of *Cryptococcus *(a basidiomycete genus from another phylum). A survey of other *Cryptococcus *species within the Tremellales has not yet yielded any further PRP8 intein sequences but has shown 'empty sites' in eight species, including in *C. amylolentus*, the most closely related species to *C. neoformans *and *C. gattii *[20].

### Phylogenetic analyses

To better understand the sporadic distribution of the PRP8 inteins we analysed the sequences of the PRP8 host proteins and PRP8 intein splicing domains. This analysis should determine if the inteins have a similar phylogeny to that of the PRP8 proteins (suggesting vertical descent of the intein) or, alternatively, have discordant phylogenies suggesting horizontal transfer. We chose to concentrate the phylogenetic analysis on the splicing domains for two main reasons. Firstly, *C. neoformans *and *C. gattii *have PRP8 mini-inteins, as do several of the euascomycetes. These inteins therefore have splicing domains but no homing endonucleases. Secondly, it has been shown that many of the yeast VMA homing endonucleases are non-functional and this will influence the rate of substitution and therefore potentially disturb phylogenetic analyses.

The phylogeny of the PRP8 proteins follows the expected organism phylogeny (Figure 4). The basidiomycete PRP8 sequences (*Cryptococcus, Coprinopsis, Ustilago *and *Phanerochaete*) fall within the fungal group, but are outside those of the ascomycete species. The euascomycete PRP8 proteins group into their respective orders, all well separated from hemiascomycete PRP8 proteins. There are no paralogs of the *PRP8 *gene present in any genome that might perturb this phylogeny.

**Figure 5:**
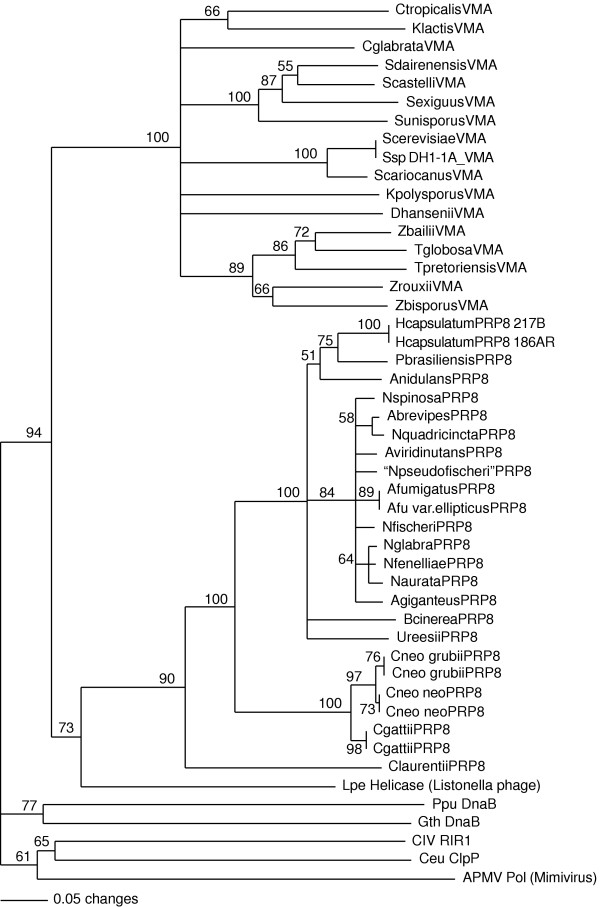
**Phylogenetic tree of inteins based on an alignment of the four protein splicing domain motifs**. Phylogenetic tree based on an alignment of the four protein splicing motifs from the PRP8 inteins and the VMA inteins, together with splicing domains from inteins found in eukaryote viruses and plastids. The tree was constructed by the neighbour-joining method using PAUP* [61] and is a consensus derived from 1000 bootstrap replicates. The numbers indicate the percentage bootstrap support (only nodes with >50% support are shown). Accession numbers of the intein sequences are as in Figures 1 and 3 or can be obtained from InBase [62]. *S. dairenensis *and *S. castellii *are now included in the newly described genus *Naumovia*; *S. exiguus *and *S. unisporus *are now included in the genus *Kazachstania *[70].

Further phylogenetic analyses were based on an alignment of the four sequence blocks/motifs of the splicing domains of the fungal inteins. These analyses indicate that the splicing domains of the euascomycete PRP8 inteins are closely similar to those of the *Cryptococcus *PRP8 inteins (Figure 5). There is much less variation between the splicing domain sequences of the euascomycete PRP8 inteins and the splicing domains of the *Cryptococcus *PRP8 inteins than there is within the splicing domains of the VMA inteins found in the single family Saccharomycetaceae. The basidiomycete and ascomycete fungi last shared a common ancestor more than 500 million years ago, while two of the most diverged VMA intein carrying species (*Kluyveromyces lactis *and *Saccharomyces cerevisiae*) diverged approximately 70 million years ago [43]. The close similarity of the basidiomycete and euascomycete PRP8 inteins does not reflect the length of time since the divergence of their host organisms, and suggests horizontal transfer from a euascomycete to a basidiomycete.

The splicing domains used in this alignment span only 126 residues and thus provide insufficient discrimination within the Fumigati group of the *Aspergillus *species to determine within-group relationships. Inteins carried by species in more taxonomically distant groups are clearly separated however (for example those in the Onygales order), indicating significant phylogenetic signal is present.

**Figure 6:**
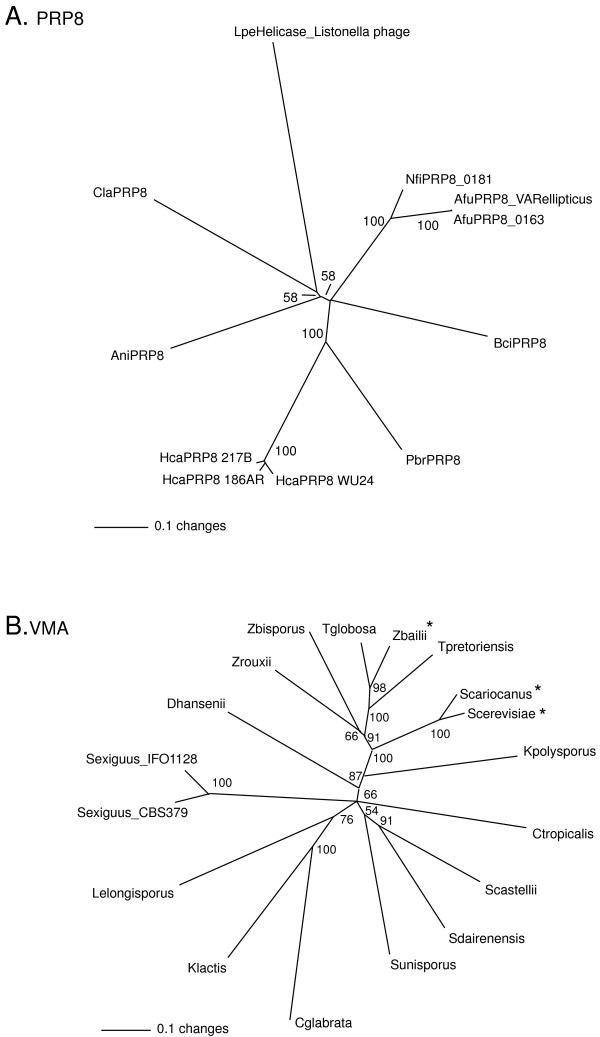
**Phylogenetic trees based on alignments of the homing endonuclease domains**. Phylogenetic trees based on alignments of the whole homing endonuclease from:A. PRP8 full-length inteins. B. VMA inteins. Asterisks denote the VMA intein homing endonucleases known to be active [9]. The trees were constructed by the neighbour-joining method using PAUP* [61] and each represents a consensus derived from 100 bootstrap replicates. The numbers indicate the percentage bootstrap support (only nodes with >50% support are shown). Accession numbers of the intein sequences are as in Figures 1 and 3 or can be obtained from InBase [62]. *S. dairenensis *and *S. castellii *are now included in the newly described genus *Naumovia*; *S. exiguus *and *S. unisporus *are now included in the genus *Kazachstania *[70].

Phylogenetic analysis of the PRP8 homing endonuclease domains was limited to the nine full-length inteins in Table 1. The phylogram (Figure 6) indicates an unresolved polytomy, reflecting the highly diverged nature of the homing endonucleases, with the exception of the closely related *Aspergillus fumigatus*/*Neosartorya fischeri *group and the moderately related *Histoplasma capsulatum*/*Paracoccidioides brasiliensis *group. This extensive divergence means that nothing can be concluded from these data about the phylogenetic relationship of the *Cryptococcus laurentii *PRP8 endonuclease with the euascomycetes. These results fit with the conclusions derived from the synonymous substitution analysis; the PRP8 intein shows great diversity. The comparable VMA phylogram also shows a great deal of diversity in the homing endonuclease domain (Figure 6), however much of this diversity is in inactive endonucleases that would be expected to evolve more quickly.

The PRP8 and VMA HEG domains show comparable protein diversity. In contrast, the synonymous substitution frequency has reached saturation in the PRP8 HEGs, while there is much less synonymous substitution present in the VMA HEGs. These observations taken together suggest that the PRP8 intein is the more ancient element.

## Discussion

In the introduction we described a model in which HEGs (and more specifically, homing endonuclease-containing inteins) undergo recurrent cycles of (i) horizontal transmission to new genomes, (ii) spread and fixation in the recipient population by homing, (iii) degeneration due to the absence of target sequences and (iv) eventual loss [11]. This general model could be applied to any HEG but the HEGs of the VMA inteins of the saccharomycetes provided much of the experimental basis for the model. Our results allow evaluation of this model in the context of a second group of nuclear inteins. Relevant gene distribution models, used to investigate patchy, non-phylogenetic gene distributions, have been the subjects of several studies [44,45].

### Full length inteins: active and inactive homing endonucleases

The majority of the homing endonucleases encoded by the VMA intein sequences are no longer functional [9]. *In vitro *assays of the activity of the VMA intein homing endonucleases have shown that only three are functional [9]. The dS/dN values derived from comparisons of these active inteins (calculated across a concatenation of the conserved motifs C, D, E and H) are higher on average than the dS/dN values produced when inactive homing endonucleases are compared. As noted by Koufopanou and Burt [36] the dS/dN value of the splicing domain is strongly positive for the VMA inteins, including those with an inactive homing endonuclease. To take one of the extreme examples, our comparison of two species with inactive VMA homing endonucleases (*S. exiguus *and *Candida glabrata*) gave dS/dN of 4.41 for the splicing domain and a dS/dN of 0.67 for the homing endonuclease domain. In other words, when inteins with inactive homing endonucleases are compared the dS/dN value of the (active) splicing domain is different from that for the inactive homing endonuclease. Comparison of the dS/dN value of the splicing domain and homing endonuclease domain of the PRP8 inteins suggest that both of these domains are constrained (both show high dS/dN values). For example, comparison of the *Histoplasma *and *Paracoccidioides *inteins show a dS/dN value of 23.57 for the splicing domain and a dS/dN value of 14.27 for the homing endonuclease domain. The euascomycete PRP8 homing endonucleases almost all contain the two critical aspartate residues of the active site. It is therefore likely that they are active although this clearly requires experimental confirmation.

Even though the PRP8 homing endonucleases appear to be constrained by selection, they are nevertheless quite diverse. This may be attributable, in part, to a long evolutionary separation but it also seems to reflect a relatively relaxed selection as compared to the splicing domain. The diversity of the homing endonucleases is shown by amino acid substitution as illustrated in the phylogenetic tree (Figure 6), but there is also frequent indel occurrence. This diversity makes the homing endonucleases useful sequences for the study of the phylogenetic relationships of intein carrying fungi. Many major human pathogens (*A. fumigatus*, *Histoplasma*, *Paracoccidioides*) and plant pathogens (*Botrytis*) carry inteins with homing endonucleases that, because of their diversity, can facilitate phylogenetic analysis.

### Mini-inteins: artificial and natural

The VMA inteins of the saccharomycetes are all full-length, homing endonuclease-containing inteins. No allelic VMA mini-inteins have been reported. Chong and Xu [13] created artificial, splicing-competent, VMA inteins by the deletion of the internal homing endonuclease region (motifs C, D, E and F) from the *S. cerevisiae *VMA intein. In order for the splicing activity to be restored in these artificial constructs, the 183amino acid residue deletion had to be replaced by a 14 or 19 amino acid residue 'linker' region. Chong and Xu [13] suggest that the linker provides sufficient flexibility to allow correct conformation for efficient splicing. This result demonstrates that a single deletion event removing all of the HEG is compatible with retaining splicing function (that is, the HEG and splicing functions are separate).

In contrast to the VMA inteins of saccharomycetes and the GLT1 inteins present in other ascomycetes [46], which are all naturally full-length, the PRP8 inteins of the basidiomycetes and euascomycetes are found both as mini-inteins and homing endonuclease-containing inteins. We previously reported that the *PRP8 *genes of *Cryptococcus neoformans *and *C. gattii *encode a mini-intein [19,20]. The full-length euascomycete PRP8 inteins vary in length from 517 to 838 residues; the mini-inteins, in contrast, are all of very similar length (153 to 180 residues), with only the *A. giganteus *and *Cryptococcus *mini-inteins being somewhat different in length. We have not detected inteins of an intermediate length or inteins with only remnants of any of the homing endonuclease domains. This raises the interesting questions of why the PRP8 system should have frequent mini-inteins and also how a full-length intein might be precisely reduced to a mini-intein.

### Empty alleles and the intein cycle

Inteins are often present at highly conserved sites in their host proteins, presumably because such inteins are less likely to be deleted. The model of Burt and Koufopanou [11] proposes that inteins can be deleted to regenerate target sites and that target sites can be invaded through horizontal transmission. The allelic PRP8 inteins are present at a highly conserved site in a highly conserved protein that forms an essential part of the spliceosome [23]. The elimination of the intein or mini-intein encoding sequence would have to be exact to allow the continued function of the protein encoded by the *PRP8 *gene. Another aspect of the model is the expectation that the frequency of the HEG allele will increase within the gene pool and eventually come to fixation. The model therefore proposes that, if a HEG is present in a species, then all members of a species should eventually carry the intein. This is not the case for *S. cerevisiae *where some members do not have the VMA intein [18; and authors' unpublished data], or for *Candida (Pichia) guilliermondii *where some members of the species do not contain the GLT1 intein [46,47]. The genome sequencing strain of *Botrytis cinerea *has a full-length PRP8 intein, while a strain isolated in New Zealand has no allelic intein. We are investigating the intein status of other *B. cinerea *strains. At present it therefore seems true that inteins are not found at fixation in many species, perhaps most species. *Aspergillus fumigatus *may have the intein at fixation but this species seems to be an asexual clone with little polymorphism. The model of Burt and Koufopanou [11] may require modification for asexual or predominantly asexual species such as *A. fumigatus*. As pointed out by these authors, the amount of gene flow within (or between) populations and species will determine how rapidly an element such as an intein will come to fixation [8,11]. The VMA intein is only capable of homing during meiosis. If this restriction applies to other eukaryote inteins one would expect asexual species to lose their inteins more rapidly than sexual species.

Liu and Yang [25] indicated that there were no allelic PRP8 inteins in some species of *Aspergillus *(*A. flavus*, *A. niger*, *A. oryzae*, *A. parasiticus*, *A. terreus*, and *A. ustus*). In this study, we have shown that there is no PRP8 intein in *A. unilateralis *(a member of the section Fumigati). There is no PRP8 intein in *A. clavatus *(a member of the section Clavati, which is the sister clade to section Fumigati) but there is a PRP8 intein in *A. giganteus *(another member of the Clavati). There are numerous other examples where an intein is found in one species but not in a closely related species (for example, *Coccidioides*/*Uncinocarpus*, *Botrytis*/*Sclerotinia*). Determination of the PRP8 intein status of further euascomycetes related to *Aspergillus *is desirable. Vertical inheritance accompanied by multiple events of intein loss may explain the present intein/mini-intein/empty allele distribution.

To determine if an intein is present in a species it is necessary to analyse more than one member of a species (this is a limitation of the data in Table 4). Similarly, before concluding that all members of a sexually reproducing fungal species carry an intein (as the model expects will be the case at fixation) it is necessary to test a significant number of isolates from diverse origins, as suggested by Burt and Koufopanou [11]. This prediction was investigated by Okuda et al. [18], who sequenced the *VMA1 *gene (and any accompanying VMA intein sequence) from 10 strains of *Saccharomyces cerevisiae*. They detected two different groups of VMA intein sequence, one group of 3 were identical to the intein sequence in strain DH1-1A and the other group of six were identical to the strain X2180-1A. The two groups were 96% identical to each other. A final strain, NKY278 (1) did not contain an intein in VMA. A further strain of *S. cerevisiae*, the natural isolate RM11-1a, does not contain an allelic VMA intein [authors' unpublished data; 48]. It then follows that not all members of the species contained an intein, even though the *S. cerevisiae *VMA homing endonuclease is active.

### Intein, mini-intein, empty allele polymorphism

It is possible that inteins do not typically come to fixation but rather achieve an equilibrium with their empty targets within the gene pool. For example, it may be that the presence of an intein is not perfectly neutral but confers a very slight selective disadvantage due to the added process of protein splicing or the occurrence of ectopic homing endonuclease cleavage. If inteins exist in equilibrium with empty target sites within a gene pool this would allow continuous selection for homing endonuclease function during extended periods of vertical transmission. If true, this would explain how an active homing endonuclease could be retained during an extended period of vertical transmission. Segregation of the empty allele (without any requirement for deletion) would also explain the frequent occurrence of species lacking the intein. This modified model may be applicable to the filamentous fungi such as *Aspergillus fumigatus *that, because of their abundant aerial mitotic conidia, have global distributions and therefore very large effective gene pools.

### Intein phylogeny and horizontal transmission

The only occurrences of an intein in PRP8 outside the euascomycetes are those found in closely related, pathogenic, varieties of *Cryptococcus neoformans *and *C. gattii *(members of the genus: *Filobasidiella*) and in the occasional pathogen *Cryptococcus laurentii*. The absence of an intein encoded in the *PRP8 *gene of species closely related to *Cryptococcus neoformans *and *C. gattii *poses an interesting puzzle as to the evolutionary origin of the immobile mini-inteins at this site in the *Cryptococcus *PRP8 protein. If the intein were inherited vertically, it might be expected to be present in all, or at least some, of the other members of the Tremellales group, whereas the only other *Cryptococcus *species in which a PRP8 intein has been detected is *Cryptococcus laurentii *[20]. This species contains a full-length homing endonuclease-encoding intein in PRP8. *C. laurentii *is a species from a different cluster of the Tremellales group than *C. neoformans *[49]. It might be suggested as a source for the *C. neoformans *mini-inteins. The intein is absent, however, from all the other Tremellales species that have been examined, including another member of the *Filobasidiella *genus (*C. amylolentus*). The common ancestor of pathogenic cryptococci may have gained the intein by horizontal transmission of an HEG containing intein sequence from another *PRP8 *gene. The presence of an allelic, HEG containing, intein sequence in the *PRP8 *gene of a group of euascomycetes suggests that this euascomycete group is a possible source of the *Cryptococcus *mini-intein.

## Conclusion

Our analyses of the PRP8 intein group have shown many points of difference between PRP8 inteins and the VMA inteins of saccharomycetes. The PRP8 intein has been found in members of two phyla (ascomycetes and basidiomycetes), while the VMA intein of yeast is restricted to several genera in one family, the Saccharomycetaceae. Many PRP8 inteins are mini-inteins, whereas no mini inteins have been found in the VMA-a site of members of the Saccharomycetaceae. Most of the VMA inteins contain an inactive homing endonuclease. In contrast, the homing endonucleases of the PRP8 inteins are apparently active (they show high dS/dN values). The phylogeny of the euascomycete PRP8 inteins provides no evidence for horizontal transfer. Comparison of the homing endonuclease domains indicates that the level of synonymous change has reached saturation (pS >0.74) suggesting that the homing endonucleases have been diverging over a substantial period of evolutionary time. Despite this extended period of vertical transmission, the homing endonucleases have apparently remained active. The VMA homing endonuclease is not saturated by synonymous substitutions (pS<0.74). This implies that the VMA intein allelic group is of more recent origin than the PRP8 allelic group, even though the homing endonuclease amino acid sequences are more divergent than those of the PRP8 inteins. The extensive divergence in the VMA homing endonucleases is attributable to their loss of function.

We submit a modified model for intein evolution that may be more appropriate for inteins present in euascomycete species. We suggest that inteins may not become fixed in a population, but that their presence/absence may be polymorphic in a particular species. This would allow continuous selection for homing endonuclease function during extended periods of vertical transmission. Segregation of the empty allele (without any requirement for deletion) would also explain the frequent occurrence of closely related species that lack the intein.

## Methods

### Intein sequencing

Isolates of species of *Aspergillus *or *Neosartorya *were obtained from Food Science Australia [50] except for *Aspergillus nidulans *strain R20 which originates from Glasgow University and *A. fumigatus *var. *ellipticus *(NRRL5109) and strains of *A. lentulus *(FH4, FH5, FH7 and FH220) which were provided by Arun Balajee of the Fred Hutchinson Cancer Research Center, Seattle. Strains were grown on *Aspergillus *nutrient agar [51] at 27°C, or 37°C for *A. fumigatus*. A strain of *Botrytis cinerea*, isolated from a vineyard in New Zealand, was provided by colleagues at Lincoln University. Genomic DNA was isolated from 50ml overnight cultures essentially using the method of Philippsen et al. [52]. Genomic DNA samples from species within the section Fumigati were supplied by Carla Rydholm of Duke University. DNA from *Paracoccidioides brasiliensis *(strain Pb18) was a gift of Professor Gustavo Goldman from the Universidade de São Paulo, Brazil.

Amplification of the intein sequence and flanking regions was accomplished with the Expand High Fidelity PCR system (Roche, Mannheim, Germany) as outlined in Butler, Goodwin and Poulter [19]. Primers were synthesised by Proligo, Singapore; the primer sequences used are shown in Table 5. Amplifications of the internal transcribed spacer (ITS) regions, including the 5.8S rRNA gene, were performed using primers ITS1 (5' TCCGTAGGTGAACCTGCGG) and ITS4 (5' TCCTCCGCTTATTGATATGC) [53]; the mitochondrial cytochrome *b *genes were amplified using the primers (Wang_E1m (5' TGAGGTGCTACAGTTATTAC) and Wang_E2rev (5' GGTATAG [AC]TCTTAA [AT]ATAGC)[54]. The resulting PCR products were purified with Qiagen columns (Hilden, Germany) prior to automatic sequencing at the Allan Wilson Centre Genome Service at Massey University [55] using an ABI 3730 DNA Sequencer.

**Table 5:** Primer sequences used to amplify PRP8 intein encoding sequences from euascomycetes.

Primer name	Relevant species	Primer sequence
Aspfum-F1	*A. fumigatus*,	5' acagatgtcatccaagc 3'
Aspfum-F2	(including	5' tgggaaagagcatgccttgc 3'
Aspfum-F4	*Afu. Var. ellipticus*	5' gaaccaggaaatggagacg 3'
Aspfum-Rv1	and	5' ttcaacggtatcgtagcg 3'
Aspfum-Rv4	*Neosartorya fischeri)*	5' acagagtgaaccgacg 3'
		
Liu_1	Fumigati spp. and	5' atgaagagcaa [tc]cc [agct]tt [tc]tggtggac 3'
Liu_2	*A giganteus*	5' gcattcgtgag [tc]tt [ct]tt [ag]aa [tc]ttcat 3'
		
Ani-Fn	*A. nidulans*	5' agcttgtcttgccaac 3'
Ani-Fs	*A. nidulans*	5' acaaagacagaccaaccaataaacggattg 3'
Ani-Ra	*A. nidulans*	5' actgttatgcagtacaacatag 3'
Ani-Rsn	*A. nidulans*	5' tctcccaggagtctcgacgctaga 3'
		
Pb_Fwd1	*P. brasiliensis*	5' catatctctggaactgtggcag 3'
Pb_Fwd2	*P. brasiliensis*	5' gcagttgggtattgacacagtgc 3'
Pb_Rev1	*P. brasiliensis*	5' gcactgtgtcaatacccaactgc 3'
Pb_Rev2	*P. brasiliensis*	5' gcgttcgtcaacttcttgaacttcat 3'
Pb_Rev3	*P. brasiliensis*	5' gctttgtgctgcctcgttaacg 3'
Para_b-F3	*P. brasiliensis*	5' gatagaagtcgcacg 3'
Para_b-Rv4	*P. brasiliensis*	5' gggtcggtttatgc 3'

General sequence analyses were done using 4Peaks V1.6 [56] and the Wisconsin GCG package (Genetics Computer Group, 575 Science Drive, Madison, Wis.). Sequence similarity searches were performed using the National Center for Biotechnology Information BLAST server [57] and at the various fungal genome sequencing project web sites (see below). Multiple sequence alignments were constructed using CLUSTAL_X at the European Bioinformatics Institute server [58] edited with Seaview [59] and shaded with MacBoxshade [60]. Phylogenetic trees were constructed using PAUP4b10 [61]. The rates of synonymous and non-synonymous substitutions within the intein HEG domains were calculated using Syn-SCAN, a program found within the Resources section of the Stanford HIV RT and Protease Sequence database [38,39].

Representatives of the newly described intein sequences in the *PRP8 *genes and flanking sequences have been assigned GenBank accession numbers AY832918-AY832926 (Table 1). Descriptions of the ascomycete PRP8 inteins have been added to those of the *Cryptococcus *inteins at InBase records [62].

### Fungal genome sequencing projects

#### Histoplasma capsulatum

Genomic sequence data were from the Genome Sequencing Center at Washington University in St. Louis [26] where two distinct strains of *H. capsulatum *(G217B and G186AR) are being sequenced, and from the Broad Institute [48].

#### Paracoccidioides brasiliensis

Data consist of a large set of *P. brasiliensis *ESTs generated by a group of laboratories in Brazil in order to gather information about the differences in yeast and mycelial transcriptomes of this pathogenic fungus [63]. Data are available via GenBank accessions CA580326-CA584263.

#### Aspergillus

Preliminary sequence data for *Aspergillus fumigatus *(strain Af293) were obtained from The Institute for Genomic Research website [64]. *Aspergillus nidulans *(strain FGSC_A4) data were available at the *Aspergillus nidulans *Sequencing Project of the Broad Institute of MIT and Harvard [48]. We examined several other fungal whole genome sequences (including those of *Botrytis cinerea*, *Uncinocarpus reesii*, *Coccidioides posadasii*, *Coprinopsis cinereus*, *Fusarium graminearum*, *Magnaporthe grisea*, *Histoplasma capsulatum*, *Neurospora crassa*, and *Ustilago maydis*) that are provided by the Broad Institute of MIT and Harvard [65]. The Génolevures website [66] provides data and search tools relating to the complete genomes of four hemiascomycetes and the initial data from random sequencing of nine other hemiascomycete species.

We also searched the database held by the Consortium for the functional Genomics of Microbial Eukaryotes (Cogeme). In this project, expressed sequence tags (ESTs) have been obtained from thirteen plant pathogenic fungi and two plant pathogenic oomycetes [67]. ESTs representing the same gene have been used to produce a single contig or consensus sequence and a BLAST facility is available at the website [68]. We also used data provided by the US Department of Energy's Joint Genome Institute [69] derived from the genome of the basidiomycete *Phanerochaete chrysosporium *(the white rot fungus).

## Authors' contributions

RP and MB conceived of the study, participated in the study design, data analysis and manuscript revision. TG participated in intein discovery, data analysis and manuscript revision. JG and MB performed the amplification and sequence analyses. MB drafted the manuscript. All of the authors read and approved the manuscript.

## Supplementary Material

Additional File 1 SNAP XY plots of the homing endonuclease encoding regions of the PRP8 and VMA inteins. These represent the cumulative behaviour of the average synonymous and non-synonymous substitutions across the coding region. The analysis was done at the SNAP site [40]. The positions of codons are indicated below the x-axis. The y-axis indicates the cumulative number of nucleotide changes causing synonymous (red) or non-synonymous (green) amino acid changes. Click here for file
