# Supplementary material for: The distribution and evolutionary history of the PRP8 intein
Source: BMC Evol Biol. 2006 May 31;6:42. doi: 10.1186/1471-2148-6-42 (PMC1508164; doi:10.1186/1471-2148-6-42)
Supplement: Additional File 1 — SNAP XY plots of the homing endonuclease encoding regions of the PRP8 and VMA inteins. These represent the cumulative behaviour of the average synonymous and non-synonymous substitutions across the coding region. The analysis was done at the SNAP site [40]. The positions of codons are indicated below the x-axis. The y-axis indicates the cumulative number of nucleotide changes causing synonymous (red) or non-synonymous (green) amino acid changes. [file 1471-2148-6-42-S1.pdf]

SNAP XY plots of the homing endonuclease encoding regions of the PRP8 and VMA inteins.

The plots represent the average behaviour of each codon for all pair-wise comparisons. This is shown on the graph as the cumulative number of synonymous (red) or non-synonymous (green) substitutions across the coding region. The analysis was done at the SNAP site [http://www.hiv.lanl.gov/content/hiv-db/SNAP/WEBSNAP/SNAP.html]
RESULT: The accumulation of non-synonymous changes occurs at a much reduced rate in the PRP8 homing endonuclease genes as compared to the VMA homing endonuclease genes

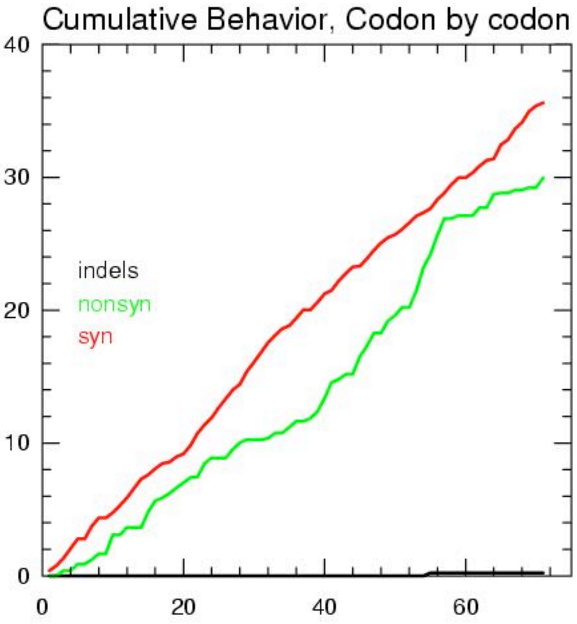

A. PRP8 homing endonuclease-encoding region.

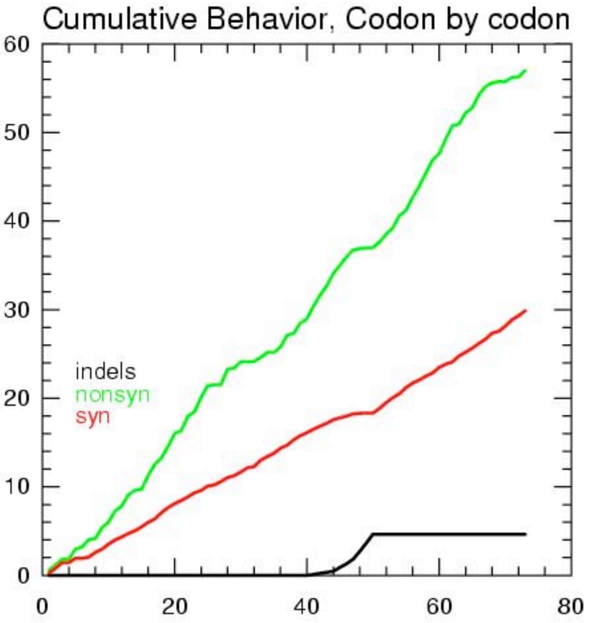

B. VMA homing endonuclease-encoding region.
